# Adventures in Semantic Publishing: Exemplar Semantic Enhancements of a Research Article

**DOI:** 10.1371/journal.pcbi.1000361

**Published:** 2009-04-17

**Authors:** David Shotton, Katie Portwin, Graham Klyne, Alistair Miles

**Affiliations:** Image Bioinformatics Research Group, Department of Zoology, University of Oxford, Oxford, United Kingdom; University of California San Diego, United States of America

## Abstract

Scientific innovation depends on finding, integrating, and re-using the products of previous research. Here we explore how recent developments in Web technology, particularly those related to the publication of data and metadata, might assist that process by providing semantic enhancements to journal articles within the mainstream process of scholarly journal publishing. We exemplify this by describing semantic enhancements we have made to a recent biomedical research article taken from *PLoS Neglected Tropical Diseases*, providing enrichment to its content and increased access to datasets within it. These semantic enhancements include provision of live DOIs and hyperlinks; semantic markup of textual terms, with links to relevant third-party information resources; interactive figures; a re-orderable reference list; a document summary containing a study summary, a tag cloud, and a citation analysis; and two novel types of semantic enrichment: the first, a Supporting Claims Tooltip to permit “Citations in Context”, and the second, Tag Trees that bring together semantically related terms. In addition, we have published downloadable spreadsheets containing data from within tables and figures, have enriched these with provenance information, and have demonstrated various types of data fusion (mashups) with results from other research articles and with Google Maps. We have also published machine-readable RDF metadata both about the article and about the references it cites, for which we developed a Citation Typing Ontology, CiTO (http://purl.org/net/cito/). The enhanced article, which is available at http://dx.doi.org/10.1371/journal.pntd.0000228.x001, presents a compelling existence proof of the possibilities of semantic publication. We hope the showcase of examples and ideas it contains, described in this paper, will excite the imaginations of researchers and publishers, stimulating them to explore the possibilities of semantic publishing for their own research articles, and thereby break down present barriers to the discovery and re-use of information within traditional modes of scholarly communication.

## Introduction

Online versions of journal articles are conventionally presented either as HTML Web pages or as static PDF documents, with the Web used primarily as a convenient distribution medium for traditional text. As the electronic embodiment of the printed page, the PDF document is both familiar and easy for humans to read, but it is antithetical to the spirit of the Web, lacking user interactivity and being difficult for machines to read, thus inhibiting the development of services that can automatically link information between articles.

Recent developments in Web technology can be used for semantic enhancement of scholarly journals articles, providing better linking to other resources, adding descriptive metadata that assist article discovery and specify the meaning of concepts and terms within the article, and allowing users access to “lively” content in the form of interactive figures, re-orderable reference lists, document summaries, and downloadable numerical datasets in which the data are both accessible and actionable.

In this paper we describe semantic enhancements made to a recently published biomedical research article from the journal *PLoS Neglected Tropical Diseases* (*PLoS NTDs*), using this single exemplar research article to create a compelling proof of the possibilities of semantic publication.

We define the term *semantic publication* to include anything that enhances the meaning of a published journal article, facilitates its automated discovery, enables its linking to semantically related articles, provides access to data within the article in actionable form, or facilitates integration of data between articles.

We undertook this exercise in semantic publishing to demonstrate some of the different types of semantic enhancement that we consider desirable for improving the usefulness of online research articles, and to show the benefits that ensue from publishing the data they contain to the Web, thus facilitating the integration of information from different publications.

### The Unique Role of the Journal Article

Since our contention is that biomedical journal articles should become better conduits for the publication of research data than they are at present, it is important to relate them to bioinformatics databases, whose primary role is clearly data publication. In 2005, Philip E. Bourne, Editor-in Chief of *PLoS Computational Biology* and Co-Director of the Protein Data Bank (PDB; http://www.rcsb.org/pdb/), wrote a stimulating article [1] in which he pointed out that the distinction between an online paper and a database was diminishing and called for “seamless integration” between papers reporting results and the datasets used to compute those results.

While we agree with his central analysis that in the electronic age the processes involved in submitting papers to journals and data to bioinformatics databases are essentially similar, we contend that similarity of process should not blind us to essential differences in purpose. Anita de Waard [2] has argued persuasively that the scientific journal article is an exercise in rhetoric, with the primary purpose of persuading readers of the truth of a particular hypothesis, and that the data it contains are carefully selected to prove that hypothesis as clearly as possible. In contrast, the function of a bioinformatics database is to provide impartial access to complete datasets. We thus believe that a clear distinction needs to be maintained between these two forms of scientific communication. The journal publication provides a peer-reviewed dated record of the authors' views at the time of publication, and as such becomes an immutable part of the scientific record, while the research database should contain the most reliable up-to-date information. Seamless integration between the two is *not* desirable. One needs to approach research publications and research datasets with different presuppositional spectacles—the first rhetorical, the other analytical—and for this reason researchers need the “seams” between the two to be very clearly visible. Nevertheless, reciprocal citation between papers and datasets is essential, and *frictionless interoperability* between papers and datasets is highly desirable.

We believe that much more can be done to make the data that *are* contained within research articles more readily accessible in machine-readable form, a sentiment that has the backing of the international scientific, technical, and medical (STM) publishing community. The 2007 Brussels declaration (http://www.stm-assoc.org/brussels-declaration/) states, inter alia, that STM publishers are committed to change and innovation that will make science more effective, and believe that raw research data, and datasets submitted with a paper to a journal, should wherever possible be made freely accessible to other scholars.

### Others' Activities in Semantic Publishing

Of course, we are not the first to suggest or implement semantic enhancements of journal articles. For example, Seringhaus and Gerstein [3] have provided a compelling vision statement concerning the optimal information architecture for biosciences publications, involving closer integration of journal text and database resources, provision of intelligent markup, and the creation of Structured Digital Abstracts (SDAs), which are machine-readable documents summarizing all the key data and conclusions of articles, including (for molecular biology articles): (a) a list of all named entities in the article (genes, proteins, metabolites, etc.), with precise database identifiers; (b) a list of the main results, described using controlled vocabularies; and (c) standard evidence codes defining the methodology by which the results were obtained [4].

This has led the editors of the Elsevier journal *FEBS Letters* to collaborate with their authors and with the curators of MINT, the Molecular INTeraction Database (http://mint.bio.uniroma2.it/), which records experimentally verified protein–protein interactions mined from the scientific literature by expert curators, to implement Structured Digital Abstracts for *FEBS Letters* papers describing protein–protein interactions [5]. These SDAs are both in the form of human readable supplements to the articles' conventional abstracts, and machine-readable XML additions to the HTML articles, containing unique protein identifiers with links to MINT and to Uniprot, the Universal Protein Resource (http://www.uniprot.org/), while drawing definitions of the types of protein–protein interaction from the HUPO Proteomics Standards Initiative's Molecular Interaction (MI) Controlled Vocabulary (http://www.psidev.info/index.php?q=node/31). Between the start of this experiment on April 9, 2008, and February 18, 2009, 90 papers were published in *FEBS Letters* with SDAs. However, while it is intended that these SDAs should be hosted along with the conventional abstracts of *FEBS Letters* by the abstracting services MedLine (http://medline.cos.com/) and PubMed (http://www.ncbi.nlm.nih.gov/pubmed/) [5], this has yet to happen.

Some publishers, notably the Royal Society of Chemistry, have taken the lead in pioneering other aspects of semantic publishing as part of their routine production schedule. Certain of their journals, for example, *Molecular Biosystems* (e.g., http://dx.doi.org/10.1039/b613673g), provide an enhanced HTML version for which semantic markup of textual terms is undertaken during the standard journal production process by skilled domain-specialist editors. This enhanced HTML version provides markup of chemical names, of terms from the International Union of Pure and Applied Chemistry (IUPAC) Compendium of Chemical Terminology—the Gold Book (http://goldbook.iupac.org/), and of semantically significant terms defined by the Gene Ontology, Cell Ontology, and Sequence Ontology. Clicking on marked-up instances links these to authoritative Web resources. For example, a chemical name links to its structural formula, a list of synonyms, its IUPAC International Chemical Identifier (InChI), an XML description in Chemical Markup Language, and patents involving use of that chemical. Similarly, a Gene Ontology term links to its definition, its GO ID number, a list of synonyms, and a list of other RSC articles referencing the term. This development, known as the RSC Project Prospect, won the 2007 ALPSP/Charlesworth Award for Publishing Innovation [6], and is thought to be the first major application of Semantic Web technologies in science publishing.

Similarly, as exemplified at http://journals.iucr.org/a/issues/2003/01/00/au0308/index.html, the subscription-access journal *Acta Crystallographica A: Foundations of Crystallography*, published by the International Union of Crystallography (IUCr), has for several years supported markup of text with terms from the Gold Book and the IUCr Online Dictionary of Crystallography (http://reference.iucr.org/dictionary/), with links to definitions.

While the importance of linking publications to research data has recently been stressed by Seringhaus and Gerstein [3] and by Borgman [7], few scholarly journals link to downloadable actionable datasets. However, there are some excellent examples of this from other sources, for example SourceOECD (http://www.sourceoecd.org/), the Online Library of Statistical Databases, Books and Periodicals of the Organisation for Economic Co-operation and Development (OECD; http://www.oecd.org/). Their online statistical tables have an “Export Excel” tab that creates and downloads an Excel spreadsheet from data currently being viewed. Users can also create dynamic graphics, bringing the data alive visually.

Despite these laudable examples, the general situation is clearly far from being resolved in terms of semantic publishing best practice, and there is considerable scope for innovative developments. For this reason, Elsevier recently launched the Elsevier Grand Challenge: Knowledge Enhancement in the Life Sciences (http://www.elseviergrandchallenge.com/), seeking proposals “to improve the interpretation and identification of meaning in online journals and text databases relating to the life sciences”, and offering substantial cash prizes for the best ideas that they could then optionally put into practice under exclusive licences. At the time of writing (February 2009), this competition is still ongoing, with four finalists, whose details are given on the Grand Challenge Web site, having been selected from the ∼70 original applicants. The Finals will be held at a scientific session at the *Experimental Biology* conference (April 18–22, New Orleans; http://www.eb2009.org/) as well as via a live free webinar on April 21, 2009, 3:00–3:30 p.m. U.S. Central Time.

### The Relevance of Our Work to *PLoS Computational Biology*

While the subject area for the work reported in this paper—infectious disease epidemiology—may not be familiar to many readers of this journal, our work of semantic enhancement to promote access to biological data within a scholarly publication by the application of computer technology falls squarely within the remit of *PLoS Computational Biology*, which has been a leader in the areas of semantic publishing and the intersection of the literature and curated databases as repositories of scientific knowledge. Furthermore, the approaches adopted in our work are of general applicability. To appreciate this, consider the case of mashups of research data with Google Maps or Google Earth, which we describe for the *PLoS NTDs* article below. While at first glance such data representations might seem far removed from molecular biology and bioinformatics, a moment's thought will show that this is not the case, as the following four examples illustrate.

1. Multi-locus sequence typing (MLST) databases established at Oxford University, Imperial College in London, and the Max-Planck-Institut für Infektionsbiologie in Berlin, record regional variants of some fifty pathogenic bacterial and yeast species, including *Helicobacter pylori*, *Escherichia coli*, *Yersinia pestis*, *Candida albicans*, and various *Campylobacter*, *Pseudomonas*, *Salmonella*, and *Streptococcus* species, responsible for a variety of serious diseases up to and including plague, and permit the locations of these variants using data mashups with Google Maps and Google Earth (e.g., http://maps.mlst.net/).
2. Current genome-wide association (GWA) studies involve scanning the genotypes of thousands of individuals to determine variation within half a million or more single nucleotide polymorphisms (SNPs), both of human patients from across the world and of their local pathogens, thereby seeking to discover correlations with disease susceptibility [8],[9]. Many of these correlations show regional localization that it would be appropriate to display using geospatial mapping.
3. The well-publicized Global Ocean Sampling Expedition of the J. Craig Venter Institute [10] (http://collections.plos.org/plosbiology/gos-2007.php) and other metagenomics projects that analyse biodiversity in various marine, freshwater, and terrestrial ecosystems across the world, are entirely dependent on geospatial metadata for organizing their results.
4. In a most elegant illustration of the interaction between genetics and biogeography (http://iphylo.blogspot.com/2007/06/google-earth-phylogenies.html), Rod Page has used Google Earth as a phylogeny viewer, displaying a phylogenetic tree for *Banza* katydids [11] as an aerial phylogram hovering over the Hawaiian Archipelago. This shows how the evolution of the insects, determined from sequence comparisons, correlates with the sequential appearance of new habitats, as volcanic islands in the chain emerged from the ocean.

## The Target for Our Semantic Enhancements

Our chosen target for semantic enhancement is the article by Reis et al. (2008) published on April 23, 2008, in *PLoS Neglected Tropical Diseases* [12] (http://dx.doi.org/10.1371/journal.pntd.0000228). The article reports studies undertaken in 2003 and 2004 of the risk of contracting leptospirosis among inhabitants of a *favela* (an urban slum settlement) called Pau da Lima, in the Brazilian city of Salvador, as determined by correlating the presence of antibodies against *Leptospira* sp. in their blood with the proximity of their homes to potential sources of infection, and with various sociological factors including income and race.

The *PLoS NTDs* article in question was an appropriate choice for our enhancement activities for a number of reasons: a) It was current, having been selected on April 30, just one week after publication, in the hope that our enhanced version of the article would be available in time to be viewed alongside the original by many readers coming to the original for the first time. b) It was in the field of infectious disease epidemiology, in which the timely availability of reliable disease incidence data that permit predictions of the severity and spread of epidemics is important, and in which we intend to invest further semantic publishing effort. c) It contained a rich variety of data types—geospatial data, disease incidence data, serological assay results, and sociological questionnaire results—presented in an interesting variety of formats—maps, bar charts, tables, graphs, and scatter plots—potentially amenable to semantic enrichments of different sorts. d) The raw data underlying these observations were not readily accessible from the original online article in actionable form. e) The article was published under a Creative Commons attribution license (http://creativecommons.org/), which meant that we were free to modify it, provided we gave appropriate attribution. f) The article was available in XML, a form that could easily be modified and republished.

The publisher and editor of the original article kindly supported our work of semantic enhancement by licensing on our behalf the additional digital object identifiers required (DOIs, administered by the International DOI Foundation http://www.doi.org/ and CrossRef http://www.crossref.org/), and by offering to provide a link to the enhanced version from the original online version of the article, and to comment on our work in the PLoS Blog (http://www.plos.org/cms/blog). Similarly, the original article's authors helped us by providing the raw data for some of their figures, by validating our citation typing, and by assisting us with Portuguese translations.

## Functional Enhancements to the *PLoS NTDs* Article

Our semantic enhancements were developed and are best seen using the tabbed Web browser Firefox v3 (http://www.mozilla.com/en-US/firefox/) running on a Windows platform. This paper will be best appreciated if the reader has both the original article (http://dx.doi.org/10.1371/journal.pntd.0000228) and our enhanced version of that article (http://dx.doi.org/10.1371/journal.pntd.0000228.x001) open simultaneously under two browser tabs, so that details can be checked, comparisons made, and enhanced interactive figures and data fusions visualized.

What we did to enhance this *PLoS NTDs* paper was not rocket science. It involved application of standard HTML markup for hyperlinks, standard use of CSS (Cascading Style Sheets) for format styling, use of simple JavaScript to provide interactivity (for example, for reference list re-ordering), use of the Yahoo! User Interface (YUI) Library of utilities and controls for building richly interactive Web applications (http://developer.yahoo.com/yui/), and use of the Google Maps API to create data fusions (http://code.google.com/apis/maps/index.html). The technical details of these enhancements are described by Shotton and Portwin in Text S1.

### Things We Did Not Alter (Much)

#### Within-document navigation

We retained all the sectional navigation links used in the original article, but moved the tabs required to activate these from the original right-hand sidebar position, where they occupied quite a lot of screen real estate, into a non-scrolling link set at the top of the document, so that they are always visible to the reader. To these navigation links we added one additional link that takes the reader to an additional Data Fusion Supplements section at the end of the article. Similarly, we retained the other pre-existing internal links: from authors' names to their institutional addresses; from in-text citations of the figures, table, and references to the corresponding items; from the figure and table thumbnails to their original full-size versions in the original article's slideshow; and from the titles of Figure S1 and Figure S2 in the main text to their original downloadable versions.

#### Reader comments

We did not duplicate the PLoS system that permits readers to record their comments, but instead encourage readers to make comments, about both the original article and our enhanced version, using the original *PLoS NTDs* system at http://dx.doi.org/10.1371/journal.pntd.0000228 and on the PLoS Blog.

### Providing Access to Actionable Data

One of the most significant aspects of semantic publication is that of making the raw numerical data contained within an article available to readers as **actionable data** that they can manipulate. A peculiar irony of the original *PLoS NTDs* article is that, while the publisher has gone to the trouble of assigning DOIs to the individual figures and the table, and has made these downloadable, they can only be downloaded as *images* (TIFF or PowerPoint format), so that the numerical data contained within them are no more accessible than in the original article, requiring manual re-keying into a spreadsheet if the reader wishes to do anything further with them.

Upon request, Dr. Albert Ko, the senior author of the *PLoS NTDs* article, and his colleagues Drs. Renato Barbosa Reis and Guilherme de Sousa Ribeiro, kindly sent us the raw data for Table 1, Figure 2, and Figure S2 in the form of Excel spreadsheets. To these we added suitable headers to identify their provenance, but otherwise left their contents unchanged. With the cooperation of PLoS who registered new DOIs for us, we then made these spreadsheets downloadable, from the “raw data” links adjacent to the thumbnails for Table 1 and Figure 2 in the enhanced article, and in the supporting information section for Figure S2, each with its individual DOI: http://dx.doi.org/10.1371/journal.pntd.0000228.t001.x001 for the raw data for Table 1, http://dx.doi.org/10.1371/journal.pntd.0000228.g002.x001 for the raw data for Figure 2, and http://dx.doi.org/10.1371/journal.pntd.0000228.s002.x001 for the raw data for Figure S2.

An additional advantage that we discovered of publishing the raw numerical datasets in this way is that they are in fact richer in information than the figures originally published in the journal article. While Figure 2 in the original article presents serological data in histogram form in terms of three categories of cross-reacting *Leptospira* serovar categories—Copenhageni, Mixed, and Other—the numerical data contained in the spreadsheet have a higher granularity, splitting the Other category into three specific serovars— Autumnalis, Canicola, and Grippotyphosa.

### Data Fusion with Information from Other Sources

We present five examples of data enrichment made possible when data from our selected *PLoS NTDs* article are combined with information from elsewhere on the Web. Although such examples are usually termed *mashups* (Wikipedia: “In Web development, a mashup is a Web application that combines data from more than one source into a single integrated tool, thereby creating a new and distinct Web service that was not originally provided by either source.”), we prefer the term ***data fusion*** to describe this type of semantic enhancement.

#### Simple geospatial data fusion

**Figure 1:**
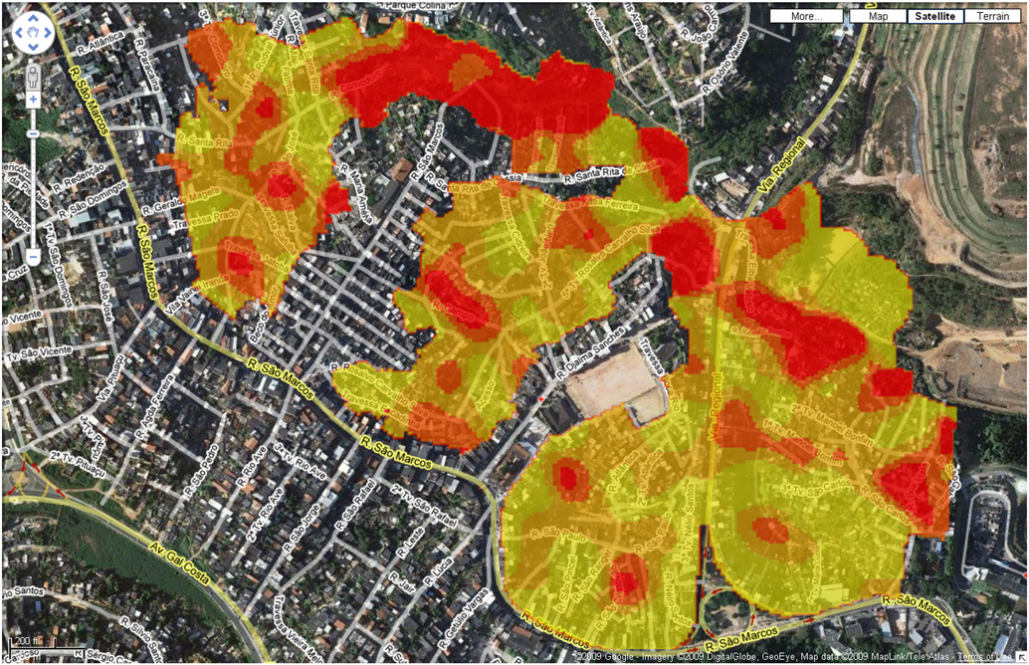
A superposition of Figure 3C of the *PLoS NTDs* article [12] onto a satellite photo of Salvador with superimposed street plan.

Panel (C) in Figure 3 in the original article is a “heatmap” showing the spatial distribution of subjects with *Leptospira* antibodies within the study area, where red corresponds to a high incidence of the leptospirosis disease. Although this is spatial information, no longitude and latitude coordinates were attached to the figure, the study location being described within the body of the article simply as “Pau da Lima, Salvador”, and shown only as a small red dot in the map inset to the article's Figure 1.

By obtaining and publishing machine-readable latitude and longitude coordinates for the study site, we were able to enhance the figure by using the Google Maps API to overlay it onto the street plan of Salvador, as shown in Figure 1 of this paper (see also the interactive zoomable version of this data fusion at http://dx.doi.org/10.1371/journal.pntd.0000228.g003.x002).

A reader viewing this enhanced version of Figure 3C has the benefit of context. For example, it is now clear that the study site is bounded by a road to the south, Rio Sao Marcos, and that there is a large building on the east side of Rio Djalma Sanches, both of which partially explain the shape of the study site. Additionally, the largest red area in the heatmap, indicating high incidence of leptospirosis, appears green with vegetation on the underlying satellite image in Google Maps (visible in the online interactive version of the figure after unchecking the check box for the heatmap overlay). This extra level of contextual information helps the reader to more quickly understand and evaluate the data being presented. Since the Google Maps mapping software is interactive, the user can zoom in and out, thereby gaining a better idea of the location of Pau da Lima within Salvador, and also the distance of the *favela* from the city centre, the coast, and other geographical features.

#### Geospatial data fusion across multiple publications

**Figure 2:**
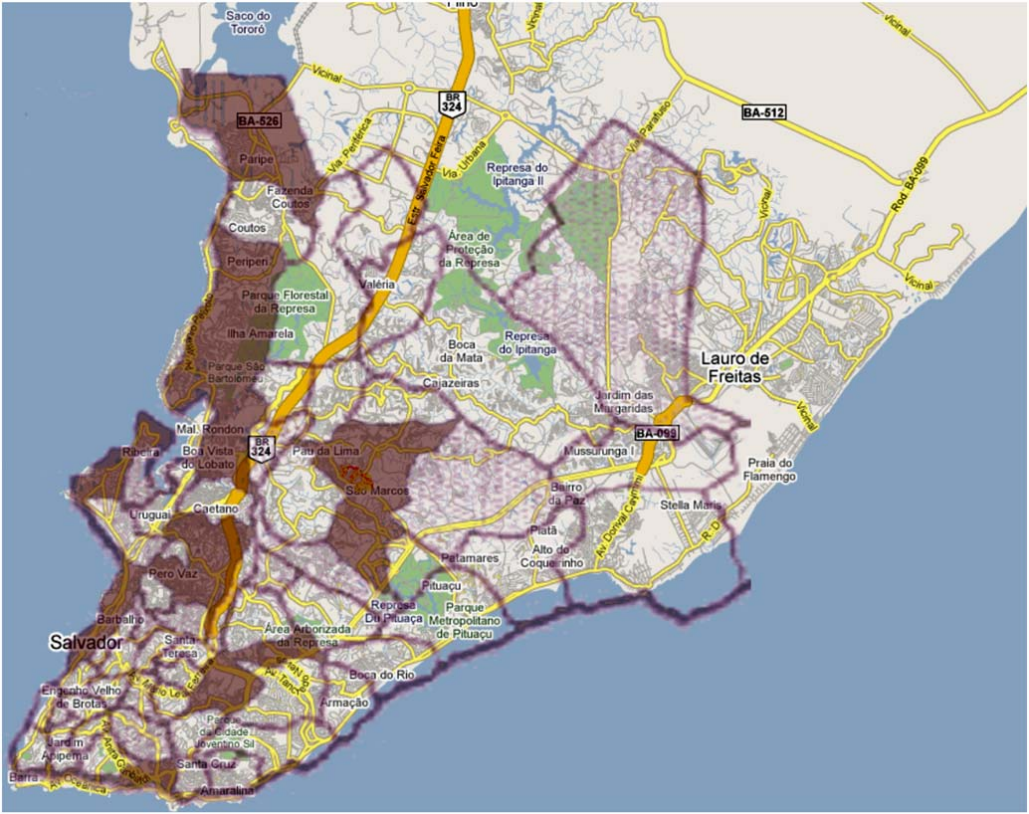
Superposition of Figure 3C of the 2008 *PLoS NTDs* article [12] and a modified version of Figure 1 from Ko et al. [13] (copyright © Albert Ko, 2008, used with permission) onto a street plan of Salvador. The study site in [12] is indicated by the small yellow-orange region in the centre of the figure.

**Figure 3:**
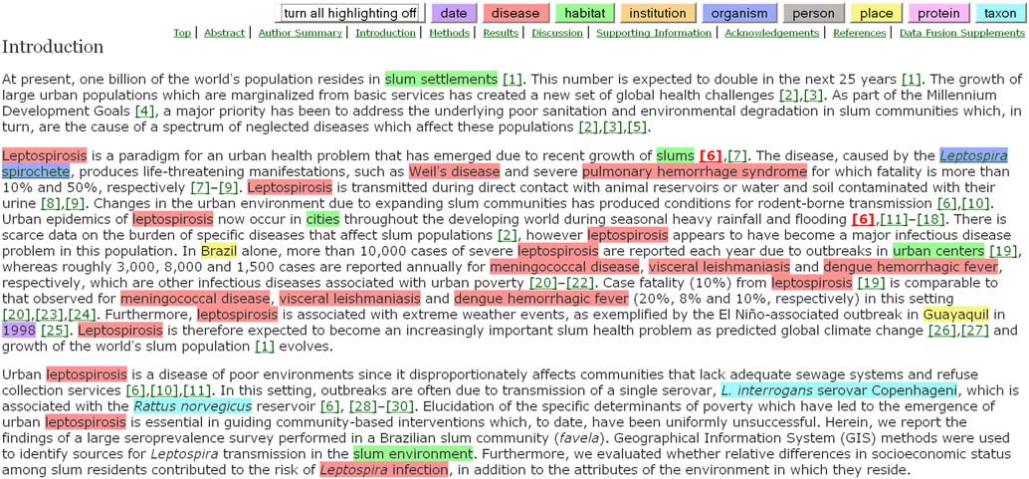
The first three paragraphs of the Introduction from the enhanced version of Reis et al. (2008) [12], with instances of all the semantic classes highlighted. The non-scrolling highlighting button set can be seen at the top of the figure, and below it the non-scrolling navigation bar.

In 1999, in the *Lancet* article [13] cited as reference 6 in Reis et al. [12], Ko and his colleagues published a map of the incidence of the disease leptospirosis across the whole of the city of Salvador, by census district. Inclusion of that 1999 map in the data fusion with Figure 3C of the 2008 *PLoS NTDs* article shows the study site in the context of the larger leptospirosis distribution in the whole of Salvador, adding value to the original figure, as shown in Figure 2 of this paper (see also http://dx.doi.org/10.1371/journal.pntd.0000228.g003.x003 for the interactive version of this data fusion).

Despite the slight misalignment of the 1999 map with Google Maps, it is clear that the small yellow-orange study site of the 2008 article in the centre of the figure lies in a census district that was reported to have a high incidence of leptospirosis, as indicated by the dark shading on the 1999 map. This suggests, for example, that the results obtained in Pau da Lima and reported by Reis et al. [12] are likely to be of relevance for other high-incidence census districts of Salvador.

#### Mapping leptospirosis study locations in space and time

Researchers studying particular neglected tropical diseases are likely to be interested in the geospatial locations and dates of others' studies on the same disease in different countries. Such information is also of vital interest to epidemiologists trying to build global models of disease prevalence and spread. To demonstrate how geo–temporal mapping might assist in this process, we determined the geospatial coordinates of a handful of recent studies on leptospirosis from different parts of the world, and indicated their relative positions on a world map by a simple data fusion with Google Maps, in a similar manner to the mappings described above. There are two variations of this data fusion, designated Data Fusions D1 and D2, referenced in the Data Fusion Supplements section at the end of our enhanced *PLoS NTDs* article.

Data Fusion D1 (http://dx.doi.org/10.1371/journal.pntd.0000228.x006) is a conventional Google Maps global mashup, in which the locations of different leptospirosis field studies in Brazil, Peru, and Thailand are displayed on a world map. The study location of Reis et al. [12] is indicated by the square red location marker. The bibliographic citations are displayed down the left side of the screen, and the location pointers of their global locations can be turned on and off at will, by selecting or deselecting the check boxes adjacent to the citations. Clicking on any one of the location pointers on the map opens an information box that gives both the location and the citation data for that study.

In Data Fusion D2 (http://dx.doi.org/10.1371/journal.pntd.0000228.x007), we added a time scale to the right margin of the map, such that the locations of studies are only displayed if their publication dates fall within the temporal range set by the two red sliders. At present, this second prototype has several limitations: it uses article publication dates rather than the dates when the reported field studies were actually undertaken, and the temporal resolution is at present only to the nearest year. Nevertheless, it serves to show the potential power of spatio–*temporal* mapping.

#### Serological data fusion across publications

We give one example, at http://dx.doi.org/10.1371/journal.pntd.0000228.x008, of data fusion that does *not* involve maps, in which we compare serological data from Table 1 of our selected *PLoS NTDs* article with similar data from Table 3 of the same research group's earlier 2008 article by Maciel et al. [14] (reference 40 in Reis et al. [12]). Both papers use a microagglutination test to detect anti-*Leptospira *sp. antibodies in the blood of subjects. This data fusion specifically concerns the age distribution of subjects showing immune responses to *Leptospira *sp. of any type. While Reis et al. (2008) [12] looked at the occurrence of *Leptospira* antibodies in a large representative population from a single slum community in Salvador, Brazil, Maciel et al. (2008) [14] looked at their occurrence among healthy members of a few “index” households of patients hospitalized with acute leptospirosis scattered across nineteen Salvador slums, and in neighbouring control households. The data fusion is presented in graphical form in the downloadable spreadsheet, as well as in the tabular form of the original sources, with explanatory text added. Since many epidemiological investigations, including the featured one by Reis et al. [12], involve long-term prospective studies or intervention trials at selected field sites or with particular cohorts of patients, this approach should also prove useful for comparing sequential studies on the same population groups.

### Adding Value to the Text

#### Highlighting of textual terms

Scholars are today faced with an avalanche of new articles that they ought to read, in excess of their available reading time. Semantic annotation of text highlights key concepts, and facilitates skimming a document in order to decide whether or not it is worth investing additional time to read it fully. We provided such semantic enhancements to the title, text, and reference titles of our selected *PLoS NTDs* article, in the form of optional colored highlighting for textual instances of nine classes of textual entities: **date**, **disease**, **habitat**, **institution**, **organism** (English name), **person** (Proper name), **place**, **protein**, and **taxon** (Latin Linnaean genus or species name), each class being associated with a particular color. In so doing, we consciously chose not to highlight terms referring to other topics, such as experimental methods.

The default setting is to have no highlighting shown, with the options either of having all the highlighting turned on, or of having one or more selected classes of terms highlighted, these options being chosen by the reader using colored selection buttons located in a non-scrolling button set at the top of the document. Figure 3 shows the beginning of the Introduction from the enhanced paper, with all the highlighting turned on. The non-scrolling navigation linkset is also visible.

Our experience in marking up the *PLoS NTDs* article clearly showed the requirement for human intervention in a process that has the potential to be automated. For example, we wished to record *slums* and *slum environments* as types of habitat in which leptospirosis was likely to occur. However, blindly marking up every occurance of phrases in which the word *slum* appeared was not appropriate, since a *slum dweller* is clearly an individual, not a habitat. To guide our markup, we developed a set of simple heuristics that may be of assistance to others undertaking similar work. These are described by Portwin and Shotton in the annotation guidelines given in Text S2.

#### Links from named entities to external information sources

In our enhanced document, most of these highlighted terms were given no external links. However, to exemplify what is possible, each instance of an **organism** in the text (i.e., *Leptospira* spirochete, rat, dog, cat, chicken, and the plural forms of these names) was given a live hyperlink to the hierarchical Linnaean classification of that species provided by the uBio taxonomic classification service (http://www.ubio.org/) (see *Leptospira* spirochete in Figure 3). This illustrates the potential power of this semantic tagging approach, whereby named entities can be linked to relevant ontological definitions or to further information, particularly valuable for readers unfamiliar with the subject matter under discussion. Linking the occurrence of named entities within the paper to resources within established controlled vocabularies is also the foundation for more precise searching over sets of papers, and for linking papers together based on commonalities in their subject matter. However, these advantages have to be balanced against the document clutter that would occur if every instance of every chosen semantic class was linked in this way. In the work we report, we avoided this tricky issue by manually linking only a single class.

#### The Supporting Claims Tooltip to permit “Citations in Context”

One reason an author cites a bibliographic reference is to provide evidence in support of his statements. In classical scholarship, thanks to the commentary tradition, citations were made to individual sections or paragraphs of referenced works, which were usually cited verbatim, since the referenced works might not be readily available to all readers. However, modern scientific references are made to the cited work as a complete entity, with no textual citation. Thus, in order to substantiate a claim, a scientific reader would normally have to leave the article currently being read and navigate to and peruse the cited reference containing the relevant evidence, leading to a significant break in concentration. To permit the key evidence to be presented to the reader in the context of the initial in-text bibliographic citation, we thus implemented a **Supporting Claims Tooltip** that permits key supporting statements from the cited reference to be displayed in a small “hover box” that appears when the reader hovers the mouse pointer over the relevant in-text reference citation. We have named this service **Citations in Context**.

Within a single article, one reference might be cited several times for different purposes. Such is the case for reference 6 in our selected *PLoS NTDs* article, which refers to the key *Lancet* article [13] that provides much of the rationale and methodological background for the subsequent *PLoS NTDs* study. That *Lancet* article is cited no less than ten times in all: five times in the Introduction, twice in the Methods, and three times in the Discussion section of the *PLoS NTDs* article. For demonstration purposes, we chose to provide Supporting Claims Tooltips for just two citations of reference 6 in Reis et al. [12] that occur in the second paragraph of the Introduction, both highlighted in red bold font in the enhanced *PLoS NTDs* article (Figure 3).

**Figure 4:**
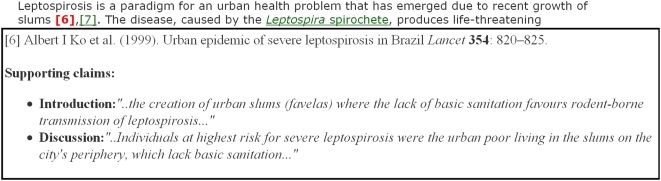
The Supporting Claims Tooltip for the first “Citations in Context” instance of the citation of reference 6 in Reis et al. [12].

**Figure 5:**
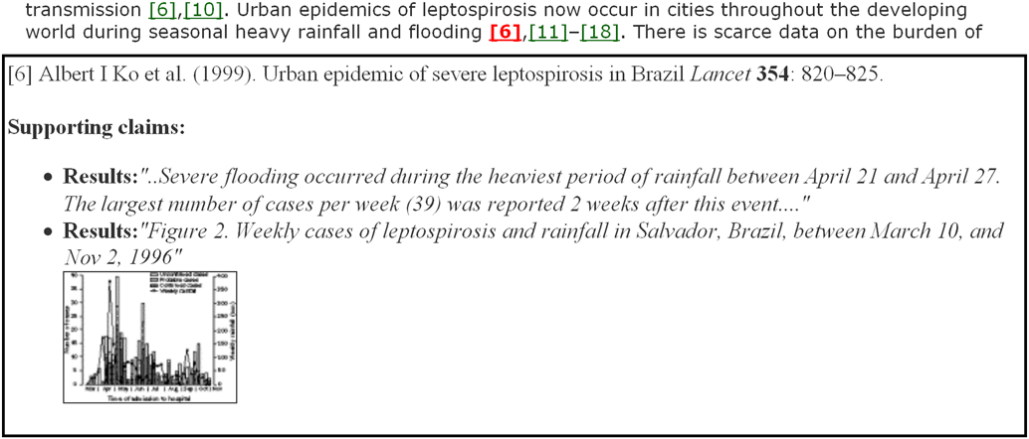
The Supporting Claims Tooltip for the second “Citations in Context” instance of the citation of reference 6 in Reis et al. [12].

The first claim, “Leptospirosis is a paradigm for an urban health problem that has emerged due to recent growth of slums [6],[7]”, is of a general character, and the corresponding Supporting Claims Tooltip, shown in Figure 4 of this paper, provides two similarly general statements from the cited *Lancet* article about the relationship of leptospirosis to slum conditions and poor sanitation. However, the second claim that cites reference 6 is much more specific: “Urban epidemics of leptospirosis now occur in cities throughout the developing world during seasonal heavy rainfall and flooding [6], [11]–[18]”. Here, hovering over reference 6 in the enhanced *PLoS NTDs* article brings up a quite different Supporting Claims Tooltip containing the information shown in Figure 5 of this paper.

In this second case, the evidence in the Supporting Claims Tooltip is taken from the Results section of the cited reference, providing real data in support of the claim in the citing article, both in terms of a textual statement relating observed rainfall and flooding to disease incidence, and by means of the caption and thumbnail image of a cited figure, showing that numerical research data exist in the cited article to back up the claim. Our Citation in Context service thus provides the reader with instant access to supporting information drawn directly from the cited work, enabling her to make an informed decision as to whether it would be worthwhile to break off from the article currently being read to peruse the cited article, to make a note to read it later, or simply to assume that the cited article does in fact support the claim made, without further need to refer to it. Since, for some readers, access to the full text of cited articles might require payment of a fee, such information, if provided for all references, would permit better use of financial resources, and might encourage fee payment for articles of proven relevance that would otherwise not have been accessed, to the benefit of publishers.

The concept of displaying a summary of the cited document, and thus giving the reader a preview of what she will see if she clicks on a link, is not new, and is often used for contextual advertising—see, for example, the Kawa demonstration at http://www.kawa.net/works/js/tips/yui-tooltips-e.html. The novel feature in our work is that the linking occurs at the level of claims, the two Supporting Claims Tooltips we implemented for separate citations of the same referenced article returning distinctly different information relevant to the context of each citation.

### Making Information More Accessible

#### Provision of a document summary

How often has a reader said “I wish I could see at a glance the most important themes and topics in this paper”, or “I wonder whether this article mentions *XXX*” (where *XXX* is a particular place, person, animal, disease, etc.)? To meet such needs, we provide a human-readable document summary (http://dx.doi.org/10.1371/journal.pntd.0000228.x002), accessed by clicking the Document Summary button immediately following the title of the article, that contains the following six sections.

***Study summary***. A simple table, specifying the disease studied, its pathogenic causative agent, principal vector, and pathogen host; the number of subjects and controls involved in the study; the indicator of infection and the assay used to detect it; the name and location of the study site, and the start and end dates of the study; and the purpose of the study and the study's principal findings. The relevance of this to the Structured Digital Abstract proposed by Seringhaus and Gerstein [3] and Gerstein et al. [4] and implemented by *FEBS Letters* [5] is discussed below.

**Figure 6:**
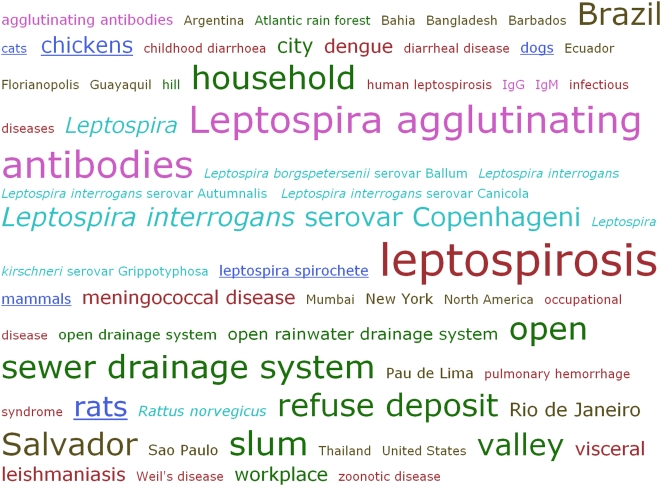
The Tag Cloud for the terms highlighted in the enhanced version of Reis et al. [12].

***Tag cloud***. The tag cloud shows, in alphabetical order, the terms highlighted in the text of the article (with the exception of institutional and personal names), displayed in their appropriate highlighting colors and with sizes proportional to their frequency of occurance in the text. Simply by running one's eye over this tag cloud, one is able immediately to see the principal topics dealt with in this article (Figure 6).

**Figure 7:**
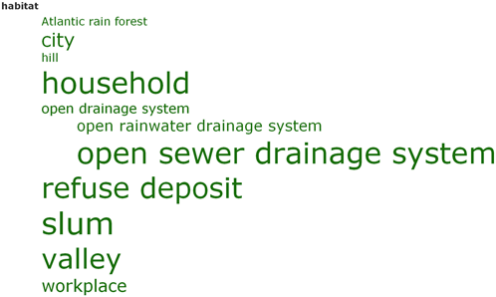
The Tag Tree for instances of the semantic concept Habitat.

***Tag trees***. Below the tag cloud, these same terms are segregated into the nine semantic classes used for highlighting the text (date, disease, habitat, institution, organism, person, place, protein, and taxon). The persons list includes both people mentioned in the text and the authors of the article, whose names are shown in bold font, but excludes the authors of cited references. In these lists, we maintained the colors and relative sizes of the terms from the tag cloud, and ordered them, where appropriate, into informal hierarchies—particularly noticeable for places and organisms. We call these displays **tag trees** (Figure 7). Tag trees provide a novel way of combining the benefits of a tag cloud with the semantic order of a hierarchy. To make the tag cloud and tag trees work effectively, we combined similar terms manually. For example, the terms “refuse”, “accumulated refuse”, “open accumulated refuse”, “refuse deposit”, “refuse deposits”, “open refuse deposit”, and “open refuse deposits” appearing in the article were amalgamated into a single term, “refuse deposit”, with an appropriate weighting.

***Infectious disease ontology terms***. Those terms relevant to the subject matter of the study by Reis et al. (2008) [12] that are present in the Infectious Disease Ontology (http://www.infectiousdiseaseontology.org) are presented as a simple list, in numerical order of their identifiers. This ontology is discussed further below.

***Document statistics***. A simple set of document statistics, summarizing the number of authors, cited references, figures, supplementary figures, and tables in the article.

***Citation analysis***. We provide a simple numerical analysis of the citations within the article, in terms of the frequency of their citation from within different parts of the document (Introduction, Methods, and Discussion). The raw actionable numerical data of this citation analysis are made available as a downloadable Excel spreadsheet from the Document Summary via a link to http://dx.doi.org/10.1371/journal.pntd.0000228.x005. This spreadsheet also contains the citation counts for the 52 cited references determined on March 11, 2009, from Google Scholar (http://scholar.google.com) and from ISI Web of Knowledge (http://apps.isiknowledge.com/).

Separate from the human-readable Document Summary, we also provide a machine-readable document information file, described below, that contains basic citation information about the chosen article.

#### Citation typing using CiTO, the Citation Typing Ontology

Conventionally, references cited in the text are simply listed in the reference list at the end of an article, without further distinction. However, added value can be given to the cited references by categorizing or *typing* both the citation itself and the cited reference. For this purpose, we developed **CiTO**, **the Citation Typing Ontology**, which provides a controlled vocabulary for the typing of citations and references. The ontology itself is available from http://purl.org/net/cito/, using content negotiation to deliver to the user either an OWLDoc Web version of the ontology when accessed via a Web browser, or the OWL ontology itself when accessed from an ontology management tool such as Protégé (http://protege.stanford.edu/). The ontology and its uses are further described in http://purl.org/NET/cito/Citation_typing_using_CiTO.doc.

In developing CiTO, we have created an ontology that should be sufficient in scope for most types of bibliographic citation encountered in scientific research articles. Authors should be able to use it to type their own citations, although there is clearly scope for the development of an ontology-backed tool to assist that process. We solicit feedback as to CiTO’s usefulness, and whether, and if so how, it should be extended.

In collaboration with the original article’s authors, we used CiTO to type the references in the selected *PLoS NTDs* article in five ways:

1. In terms of **the nature or type of the citation relationship** between a citing work and a cited work, e.g., *refutes* or *usesMethodIn*. These relationships are *Object Properties* in CiTO, and are not mutually exclusive—a single citation can have several different relationships, both factual and rhetorical.
2. In terms of **the nature or type of the work**: e.g., *Research Paper* or *Review*. These are *sub-classes of Work* in CiTO. A work should be assigned to one of these sub-classes.
3. In terms of **the nature or type of the expression** of a work: e.g., *Journal Article* or *Book*. These are disjoint *sub-classes of Expression* in CiTO. An expression should be assigned to one of these sub-classes.
4. In terms of **the nature or type of the manifestation** of an expression of a work: e.g., *PrintDocument* or *WebPage*. These are *sub-classes of Manifestation* in CiTO, and are not mutually exclusive—a single expression can have several different manifestations.
5. In terms of **the peer-review status of the expression** of a work: either *PeerReviewed* or *NotPeerReviewed*. These are disjoint *sub-classes of Status* in CiTO.

**Figure 8:**
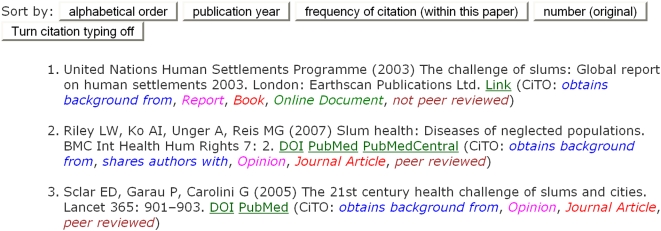
The first three references from the reference list of the enhanced version of Reis et al. (2008) [12], with the citation typing display turned on. Above the references are buttons to re-order the references, and to turn off the citation typing display.

In the enhanced article's reference list, these citation typings are not displayed by default, but may be revealed by clicking the “Turn citation typing on” button that precedes the reference list. Figure 8 shows the first three references from the enhanced *PLoS NTDs* article with citation typing turned on. The terms used in these human-readable annotations correspond to the labels of the object properties and classes within CiTO.

CiTO also permits one to record the number of times a reference has been cited by others, as determined from Google Scholar (http://scholar.google.com/) and/or from the ISI Web of Knowledge (http://www.isiwebofknowledge.com/), on a particular date, these citation counts providing proxy estimates of the global importance of each cited paper. As discussed below, we have published a separate machine-readable reference list of all the references cited by Reis et al. [12], marked up with these citation typings, and with their Google Scholar and ISI Web of Knowledge citation counts recorded.

#### Alternative language abstract

The 2008 *PLoS NTDs* article by Reis et al. [12] reports studies undertaken in Brazil with Brazilian authors, and was published with a Portuguese version of the abstract that is available within the Supplementary Information of the original article as a downloadable Word document. We converted this into a Web document, and added buttons to permit the highlighting of named entities within it, as in the main enhanced article, e.g., “ratos” and “galinhas” (rats and chickens) as organisms, and “anticorpos contra *Leptospira*” (*Leptospira* antibodies) as proteins. Furthermore, we added appropriate provenance information, including the full citation of the parent article and a Portuguese translation of its title. We assigned a DOI to this enhanced Alternative Language Abstract (http://dx.doi.org/10.1371/journal.pntd.0000228.s003.x001) and moved the link to it to a more prominent position immediately following the English language abstract, near the beginning of the article.

#### Provenance information

Frequently, Web documents contain no information concerning their origin and provenance, except for the title and authors' names, making them difficult to relocate or cite correctly. While this is not the case for journal articles, it is frequently the situation for their supplementary information files, as in the case of our chosen *PLoS NTDs* article. Here, the downloadable figures, table, and alternative language abstract all lacked any internal reference to their article of origin. Those elements of the original article that we did *not* modify, namely the article's downloadable figures and table accessed using the original PLoS DOIs, still suffer from this problem. However, to each item we modified in some manner—the interactive versions of figures, the enhanced Portuguese abstract, the downloadable spreadsheets, etc.—we added a provenance statement detailing the document to which it relates.

### User Interactivity—The “Lively” Journal Article

#### Interactive figures

One scientific conclusion of the *PLoS NTDs* article is that the risk of leptospirosis increases with increasing proximity of people's slum homes to open sewers in the bottoms of the valleys that are prone to seasonal flooding, and to open refuse deposits inhabited by rats, since these animals are the primary disease vectors, excreting the infectious spirochetes in their urine. Figure 3 of the original article (http://dx.doi.org/10.1371/journal.pntd.0000228.g003) communicates these results visually: panels (A), (B), and (C) are “heatmap” diagrams related to leptospirosis incidence, in which hotter colors indicated higher incidence; panel (D) is a topographic map; panel (E) reveals the locations of open sewers and rainwater ditches; and panel (F) highlights the sizes and locations of refuse deposits. Each panel corresponds to the same spatial area—the study site. However, it is left to the reader to overlay these maps mentally in order to obtain the visual correlations the article describes, a non-trivial task. Our enhanced version of Figure 3 (http://dx.doi.org/10.1371/journal.pntd.0000228.g003.x001) is interactive, permitting the user to drag the individual panels of the figure and superimpose them on one another at will. This greatly assists the reader in quickly obtaining a fuller understanding of the results presented, and for us this actually raised new research questions.

**Figure 9:**
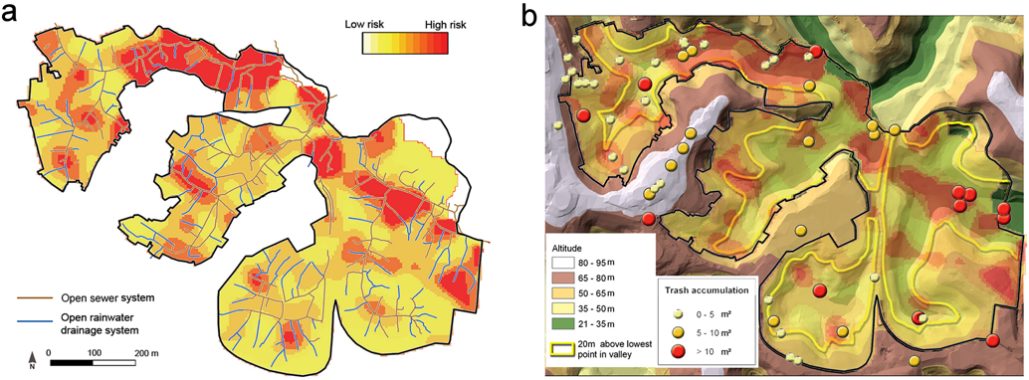
An overlay (a) of panels (C) and (E), and (b) of panels (C), (D), and (F) from the interactive version of Figure 3 of the *PLoS NTDs* article by Reis et al. [12].

For example, superimposing the locations of the sewers and ditches (Figure 3E of the *PLoS NTDs* article) on the disease incidence heatmap (Figure 3C of the *PLoS NTDs* article) clearly shows the perimeter of the study site, within which the expected relationship between high rates of disease and proximity to flood-prone open sewers in valley bottoms can be observed, as shown in Figure 9A of this paper. However, superposition of the locations of the refuse deposits (Figure 3F of the *PLoS NTDs* article) on *both* the disease incidence heatmap (Figure 3C) *and* the topographical map (Figure 3D) reveals something that was not immediately apparent when we first viewed these images side by side, although now quite obvious in hindsight. As shown in Figure 9B, this superposition shows that there is a row of medium-sized refuse deposits (yellow circles) along the light-grey crest of the hill in the left centre of the composite figure. One is immediately drawn to ask about the incidence of leptospirosis among inhabitants of that region, far from open sewers, close to refuse deposits, and outside the study area.

#### Optional re-ordering of the reference list

The original reference list in the *PLoS NTDs* article is given in numerical order, the order of citation throughout the text of the document. However, it might sometimes be useful for the reader to be able to view the reference list in other ways, of which the most obvious is alphabetical order. We added an array of buttons immediately before the reference list that gives the reader the ability to re-order the references in four ways: in **alphabetical order**, by **publication year**, by **frequency of in-text citation**, and by **reference number** (i.e., reversion to the original order) (Figure 8). When the third option is selected, the references are displayed with a font size proportional to their frequency of citations within the text, as in a tag cloud, with reference 6 in Reis et al. [12] (the 1999 *Lancet* article by Ko et al. [13]), which is cited ten times in the text of the *PLoS NTDs* article, topping the list in large font.

One additional aspect of Citations in Context, discussed above, also involves reference re-ordering. Clicking on the selected reference 6 in each instance in which the Supporting Claims Tooltip was implemented (“[6],[7]” in the first instance; “[6], [11]–[18]” in the second) takes the reader, as expected, to reference 6 in the reference list at the end of the document. However, in these particular cases the displayed references are, for the reader's convenience, slightly re-ordered. In the first instance, references 6 and 7, which are cited together, are slightly separated from the preceding and subsequent references, while in the second, reference 6 is immediately followed by the other references cited in this context, i.e., references 11–18, so that the reader might more easily check their relevance, these again being slightly separated from the preceding and subsequent references.

### Provision of New Hyperlinks

Fundamental to the Web is the ability of the reader to move from one Web page to another using hyperlinks. However, the original version of our selected *PLoS NTDs* article is sadly devoid of such external hyperlinks, severely limiting the reader's ability to discover more about items referred to in the text. We therefore added conventional Web hyperlinks wherever these are likely to be of interest or help to readers:

#### Links to cited references

If there is one thing that a reader expects from an online journal article, it is direct links to the references cited by that article. To us, the most surprising aspect of the original *PLoS NTDs* article was that its reference list failed to provide these. Despite the fact that 28 of the 52 references cited by the authors had been assigned digital object identifiers (DOIs), only two were given in the reference list (for references 10 and 40), and neither of these were in the form of active hyperlinks. Instead, PLoS provides a generic link from each reference, labelled “FIND THIS ARTICLE ONLINE”, that takes the reader to a page stating “The article may exist at PubMed/NCBI or Google Scholar”, permitting her to try searching either! The reader of the original *PLoS NTDs* article is thus at least two clicks away from the abstract or full text version of any referenced article, *and* faced with a cognitive decision of whether to use PubMed or Google Scholar to find it, making this a highly inefficient system and a significant barrier to scholarship.

We rectified this situation in the enhanced article by providing the 28 journal article references to which DOIs has been assigned with hyperlinked DOIs that resolve directly to the referenced articles (e.g., for reference 2 in Reis et al. [12]: http://dx.doi.org/10.1186/1472-698X-7-2 resolving to http://www.biomedcentral.com/1472-698X/7/2), allowing the reader direct access to this article (provided it is Open Access or her institution has a subscription). For the first few journal references, as a demonstration of principle, we also provided direct links to their abstracts in PubMed using the references' PubMed IDs and to open access full-text copies in PubMed Central (e.g., for reference 2 in Reis et al. [12]: PubMed http://www.ncbi.nlm.nih.gov/pubmed/17343758; PubMedCentral http://www.pubmedcentral.nih.gov/articlerender.fcgi?tool=pubmed&pubmedid=17343758). Three references (references 12, 47, and 49) were to journals that do not use DOIs, and for these we provided PubMed or direct Web links. For the three book references (references 41, 42, and 51), we provided links to the appropriate publishers' book pages. Eighteen of the references cited in our chosen article were to official reports from various Brazilian or international agencies that lacked DOIs: for the fourteen that were available online we provided direct Web hyperlinks.

#### Hyperlinks to external sites

We also added links to the home pages of the authors' academic institutions, of their funding agencies and software suppliers, and of the various infectious disease research centres and government agencies cited in the article. In our new document header, we added links to the original article and to the home page of *PLoS NTDs*; in the document footer we added links to Nature's citation bookmarking service Connotea, to the Web 2.0 social bookmarking service Delicious, to our own Creative Commons license for the enhanced work, and to the XHTML/RDFa Web page validation service of the World Wide Web Consortium (W3C) that shows that our enhanced work meets these standards for Web interoperability. In the article's citation box below the Author Summary, we added a link from the pre-existing PLoS copyright statement to the Creative Commons Attribution License under which the original article was published. We also added an enhancement citation box directly following the article's own citation box, which contains a similar link to the Creative Commons Attribution License for the enhanced work, and a link to our own Image Bioinformatics Research Group (IBRG) home page (http://ibrg.zoo.ox.ac.uk/).

### Machine-Readable Citation Metadata

Machine-readable metadata are central to the creation of a Semantic Web of interoperable linked data [15]. We addressed the issue of machine-readable citation metadata for our enhanced version of Reis et al. [12] in three ways: by embedding the article's self-referencing and provenance metadata within the document itself, by publishing a separate file giving this information in more detail, and by publishing a second file giving the article's annotated reference list and citation typings. While the first two of these involves considerable duplication, we wished to illustrate both methods as a guide to others.

#### Embedded machine-readable metadata—Use of RDFa

RDF, the Resource Description Framework (http://www.w3.org/RDF/), is the standard Semantic Web knowledge representation language used to conveys meaning about the relationships between Web resources, and forms the basis for the Web Ontology Language OWL (http://www.w3.org/2004/OWL/). The RDFa standard (http://en.wikipedia.org/wiki/RDFa; http://www.w3.org/TR/xhtml-rdfa-primer/; http://www.w3.org/TR/rdfa-syntax) provides a method for embedding RDF metadata statements into conventional HTML Web pages. Using RDFa, we embedded into the enhanced *PLoS NTDs* article metadata concerning the article's citation, the authors, the languages used (English and Portuguese), the DOI, the Creative Commons Licence under which it was published, and the geo-coordinates and dates of the study. While primarily intended for automated processing, the RDFa can be downloaded for human inspection using the “Extract RDFa” link in the enhanced document's footer.

#### Machine-readable self-referencing metadata—The Notation 3 document information file

Self-referencing details are also given in a separate RDF document, available from http://dx.doi.org/10.1371/journal.pntd.0000228.x003, in Notation3 format (N3; http://www.w3.org/DesignIssues/Notation3.html, http://www.w3.org/2000/10/swap/Primer.html). This contains the same information as the embedded RDFa, plus the citation typing of the article itself, and the article's abstract. The relevance of this to the Structured Digital Abstract proposed by Seringhaus and Gerstein [3] and Gerstein et al. [4] and implemented by *FEBS Letters* [5] is discussed below.

#### Machine-readable reference list

A separate machine-readable RDF document containing all the information from the typed reference list of the enhanced *PLoS NTDs* article, together with the citation counts for the cited articles, is available from http://dx.doi.org/10.1371/journal.pntd.0000228.x004. This also uses Notation3 format.

In creating these machine-readable metadata, we used namespaces that are simple, relevant, and widely used within the metadata community: Dublin Core (DC; http://dublincore.org/) and DC Terms (http://dublincore.org/documents/dcmi-terms/) for basic metadata, Friend of a Friend (FOAF; http://www.foaf-project.org/) for personal information, PRISM (http://www.prismstandard.org/) and Functional Requirements for Bibliographic Records (FRBR; http://www.ifla.org/VII/s13/frbr/frbr.htm) for selected publishing and bibliographic terms, and standard time tags (http://www.w3schools.com/tags/html5_time.asp) and geo tags (http://geotags.com/geo/geotags2.html) for temporal and geographical data. Where we could find no appropriate external ontology, as was the case for citation typing, we created one, namely the Citation Typing Ontology (http://purl.org/net/cito/) described above.

### Enhancements We Did *Not* Implement

In undertaking the work described in this paper, we first scoped possible enhancements, identifying those that were easy, moderately difficult, or hard to implement, and those that were essential, desirable, or peripheral to our primary purpose of providing a compelling “existence proof” of the possibilities of a semantically enriched publication. Within the limited resources available for this unfunded project, we then implemented all those enhancements that were easy, all those that we judged to be essential whatever their difficulty, and most of those that were desirable but moderately difficult. Features we wished to implement, but for which we did not have time and resources, or that were impossible for technical or ethical reasons, are noted here.

#### Datasets

Publication of numerical data was not undertaken for Figure 1 (maps and photographs), Figure 3 (spatial distribution diagrams and topographic map), Figure 4 (graphical output from a modelling program), or Figure S1 (further spatial distribution diagrams) of the *PLoS NTDs* article, either because these figures did not contain numerical data or because such publication would not help readers lacking access to the specialist geospatial and modelling software the authors used to manipulate these ArcView and R data files. For reasons of subject privacy and patient confidentiality, it would not have been appropriate to publish the entire database of sociological, geospatial, and serological information collected by the authors of the *PLoS NTDs* article during their extensive studies, since mining of that database could disclose the identity of individuals. Nevertheless, our publication of the three spreadsheets for Table 1 and Figure 2 and Figure S2 illustrates the principle of providing **actionable data on the Web**.

#### Semantic lenses

Ideally, mousing over one or other of the diagrams in the article would permit various semantic lenses to return numerical data to the user: for example, for the histograms of serological data in Figure 2 of Reis et al. [12], the numbers underlying each histogram bar; or for the spatial distribution diagrams in Figure 3, the distance of a household from the nearest refuse deposit or open sewer, and its spatial coordinates (latitude and longitude). Other types of semantic lens, for example, showing images of the same region before and after a tsunami (http://gis.esri.com/library/userconf/proc05/papers/pap1628.pdf), have been demonstrated elsewhere but are not relevant to our selected *PLoS NTDs* article.

#### Highlighting of semantic concepts in hues suitable for color-blind people

For this published demonstration, we did not implement alternative color palettes that would better suit readers with differing forms of color blindness (http://en.wikipedia.org/wiki/Colorblind#Types), although that would be possible, ideally as a customizable option.

#### Structural markup of greater granularity

We retained but did not extend the existing *PLoS NTDs* practice of providing structural markup of and links to different sections of the article (e.g., http://dx.doi.org/10.1371/journal.pntd.0000228#s4 for the Discussion section). However, citing particular claims from other articles, as for our Citations in Context service, would be facilitated if text was structurally marked up with machine-readable code to the level of the paragraph, the sentence, or even the individual word (all perfectly feasible in the hidden XML code behind the displayed human-readable document), or if particular rhetorical elements (hypotheses, claims, supporting statements, refutations, etc.) were marked up as such, following the suggestions of de Waard et al. [2]. Ideally, such structural markup should conform to the National Library of Medicine Document Type Definition (NLM DTD; http://dtd.nlm.nih.gov/publishing/), as defined by the NLM Journal Publishing Tag Set Tag Library version 3.0 at http://dtd.nlm.nih.gov/publishing/tag-library/3.0/index.html, since this is becoming the de facto standard for in-house journal production, such that NLM DTD structural markup is already a component of the pre-publication XML versions of many journal articles.

#### Citation network analysis

While our limited data fusion examples demonstrate the potential of integrating information between publications, and the value of imparting geo-coordinates to articles and their spatial figures so that they can be mashed up with Google maps, much more is possible. One area we are keen to explore, but in which we are frustrated by the lack of freely available metadata, is that of **citation network analysis**, for which the prerequisite is free access to machine-readable reference lists, of the type provided for our own enhanced article at http://dx.doi.org/10.1371/journal.pntd.0000228.x004. There have been numerous theoretical studies of citation networks using computer science citation data available in CiteSeer (http://citeseerx.ist.psu.edu/). However, there are no citation network analysis or visualization tools that research biologists actually use in their day-to-day research to assist them, in this age of information overload, more quickly to overview or navigate their literature, or to find key papers in new areas of interest. Such tools would enable readers to see the degree of connectedness of one article with others, and to assess its significance in the whole ecosystem of publications in the particular domain. They would also permit differing semantic views to be generated through the citation network, for example, showing only other studies on the same disease, in the same geographical location, or using the same analytical techniques.

## Ontologies and Metadata Standards for Infectious Disease Epidemiology

### The Need for Standards

Descriptive metadata in the form of controlled vocabularies or ontologies that can be used to describe accurately biological entities and experimental findings are widely recognised as key for effective resource discovery and data integration. The molecular biology community has led the way in this area, with the development and widespread adoption of the Gene Ontology (http://www.geneontology.org/). In a related more recent development, the Genomic Standards Consortium (http://gensc.org/gsc/gcat) is working toward the development of controlled vocabularies for describing complete genomes and metagenomic datasets, of great relevance to the computational biology community.

There are immense semantic challenges in capturing metadata in such principled ways: first in developing terminologies that are sufficiently comprehensive, usable, and stable that people will actually employ them to annotate their research data; and second (and more difficult) in keeping them up to date in the face of evolving biological knowledge, in ways that permit provenance records and interpretation of legacy metadata. This requires the involvement both of ordinary “bench” practitioners in each particular research area, to ensure that the artefacts developed meet real user needs, and of specialists skilled in knowledge management technologies, to ensure that the ontologies are well-formed and interface gracefully with other pre-existing ontologies without duplication of concepts. In this work, the coordinating work of OBO, the Open Biomedical Ontologies movement (http://www.obofoundry.org/), is of vital importance.

Within the biomedical community, this issue of metadata standards is particularly being addressed by the Semantic Web Health Care and Life Sciences (HCLS) Interest Group of the World Wide Web Consortium (http://www.w3.org/2001/sw/hcls/), of which our research group, IBRG, is a participating member. Significant advances in making bioinformatics data available as linked data has been made by the Bio2RDF Project (http://bio2rdf.org) [16]–[19]. IBRG is also participating in the development of VoiD (Vocabulary of Interlinked Datasets), an RDF-based schema to describe linked datasets (http://semanticweb.org/wiki/VoiD). Separately, we have proposed the use of RDF Named Graphs for provenance tracking in linked data [20],[21].

### The Infectious Disease Ontology

To date, there has been relative lack of effort in developing metadata standards in the infectious disease domain, which is a major stumbling block for epidemiology research and biosurveillance data collection activities. To address this problem, the Infectious Disease Ontology (IDO) consortium (http://www.infectiousdiseaseontology.org/) is working to develop both a core ontology for infectious disease in general, and more specialist ontologies for different subdomains of the infectious disease field, including specific diseases such as influenza, malaria, and tuberculosis, and specific research areas such as vaccine development. Terms from the IDO core ontology relevant to the work described by Reis et al. in their *PLoS NTDs* article have been included in our Document Summary of that publication, described above. Separately, Biocaster (http://www.biocaster.org), a global health monitoring service based in Tokyo, has developed a multilingual ontology suitable for describing epidemics [22] that it uses as the basis for its text-mining system for detecting and tracking the distribution of infectious disease outbreaks from information on the Web harvested from >1700 multilingual RSS news feeds [23]. Here, new text-mining methods involving role-based filtering of disease outbreak reports are proving highly effective in distinguishing reports relevant to disease outbreaks from those that are not, prior to more detailed semantic analysis, with F-scores (combining precision and recall values) in excess of 90% [24]. However, leptospirosis is not among the diseases so far addressed by either of these developments.

### Developing a MIIDI Standard—Minimal Information Required for Reporting an Infectious Disease Investigation

Conducting systematic reviews of medical research findings requires the ability to search for research articles matching precise criteria, for example, studies in which the number of patients was in excess of 1,000, in which age- and sex-matched controls were included, in which the studies were conducted between certain dates, or where the assay for the disease involved positive identification of the infectious organism in the blood of the patient, rather than simply the presence of antibodies against that organism. Such systematic reviews are currently conducted manually by experts, such as those working within the Cochrane Collaboration (http://www.cochrane.org/; http://www.thecochranelibrary.com). Clearly their work, and similar data integration activities, could be greatly facilitated if articles reporting field studies of infectious diseases were accompanied by machine-readable metadata files specifically defining the criteria used in the investigation and the main findings, along the lines of the human-readable Study Summary information contained in our enhanced article's Document Summary (http://dx.doi.org/10.1371/journal.pntd.0000228.x002).

Several minimal information standards have recently been developed as part of the MIBBI Project (Minimum Information for Biological and Biomedical Investigations; http://www.mibbi.org/) [25], each for a different type of investigation. The Structured Digital Abstracts currently used by *FEBS Letters* are based on one of these, the MIMIx standard (minimum information required for reporting a molecular interaction experiment; http://www.mibbi.org/index.php/Projects/MIMIx), which was developed by a large community of experts in the protein interaction field [5].

The Study Summary that forms part of our Document Summary described above has much in common with such minimal information standards, although it relates only to the single *PLoS NTDs* article we chose to enhance and is not presently in a machine-readable format. What is now required is for the community of infectious disease researchers collectively to develop a **MIIDI Standard—minimal information required for reporting an infectious disease investigation**. Although almost all the existing minimal information standards registered with MIBBI relate to laboratory-based bioscience investigations, it is to be hoped that MIIDI could become one of the MIBBI Standards, thereby benefiting from the considerable work this standards community has already undertaken. The MIIDI Standard, once developed, could then form the basis for the development, in collaboration with publishers, of machine-readable Structured Digital Abstracts for infectious disease journal articles, that would be of enormous value to those wishing to integrate information across such publications for systematic reviews and other purposes. We propose to host a meeting for the purpose of developing the MIIDI Standard in September 2009, and to invite interested parties to contact the senior author of this paper (david.shotton@zoo.ox.ac.uk) for further information.

## Cost-Benefit Analysis of Semantic Enhancements

The semantic enhancements applied to our selected *PLoS NTDs* article required about ten person-weeks of effort, most of which was taken in understanding, deciding, and prototyping exactly what to do and how best to do it, since this was a new area of endeavour for us. Were we to repeat the exercise with our present experience, it could be accomplished in a small fraction of that time.

While, for the purpose of this demonstration, we undertook the work manually and post-publication, the key questions this work raised are whether the added value achieved was worth the effort invested, how fast these enhancements could be brought into mainstream STM journal publishing in an affordable manner, and the degree to which semantic enrichment could be automated. We are neither publishers nor economists, and must leave it to others better qualified to judge the practicalities of implementing the proposed enhancements in an affordable and sustainable manner across a publisher's suite of journal titles.

However, we are encouraged to note that two of the finalists in the Elsevier Grand Challenge are developing automated systems that closely resemble two aspects of what we independently implemented manually. One team from the European Molecular Biology Laboratory in Heidelberg has developed a free service called **Reflect** (http://www.elseviergrandchallenge.com/team1.html, http://reflect.ws) that can be installed as a plug-in to Firefox or Internet Explorer. With a single mouse click, Reflect will tag the names of genes, proteins, and small molecules contained in any Web page, usually within a few seconds. Clicking on a tagged item will then open a pop-up window showing a concise summary of important features, such as sequence (for proteins) or 2D structure (for small molecules), and allowing navigation to commonly used source databases such as Uniprot. Another team, from Macquarie University and the CSIRO ICT Centre in Sydney, have developed a service that they call **citation-sensitive in-browser summarisation of cited documents** (http://www.elseviergrandchallenge.com/team5.html), that uses text matching between the context of a literature citation in an online paper and related sentences within the cited paper to achieve an on-the-fly automated version of the Citations in Context service described above.

### Publishers' and Editors' Roles in Semantic Publishing

In a separate paper [26], we reviewed the status of semantic publishing in the autumn of 2008, and the prospect for such semantic enhancements becoming routine. In brief, we see publishers, editors, and authors all playing important roles.

In subscribing to the Brussels Declaration, STM publishers have already aligned themselves with the aims of semantic publishing, and are seeking ways to implement these commitments in an affordable manner. So what data should the publishers make freely available? Minimally, they should provide machine-readable provenance information about the article itself, and ideally a fully featured domain-specific Structured Digital Abstract. The datasets that underlie the figures and tables in their articles should be made accessible in actionable form, and machine-readable reference lists should be made available so that citation networks can be created, analysed, and used to promote reader traffic to both citing and cited articles, to the mutual benefit of the publishers concerned. The Royal Society of Chemistry's Project Prospect described above, which relies heavily upon the domain expertise of its editors for the creation of textual semantic markup, provides a paradigm for using the skills of specialist editors to provide semantic enrichment to journal articles.

### Authors' Roles in Semantic Publishing

Authors know better than anyone else their domains of discourse, and the position of their articles within them. Only the authors really know why they cite particular papers in preference to others, and the nature of both the citations and the cited articles. If that tacit knowledge could be captured using a reference annotation tool, employing terms from the Citation Typing Ontology, the work of developing typed reference lists would essentially be done.

The Microsoft Corporation has recently released Version 1.0 of a plug-in for MS-Word 2007 (http://tinyurl.com/5szjly) for the creation of *structural* markup that supports the National Library of Medicine's document format used by many publishers and by PubMed Central. Additionally, Microsoft has just published a second plug-in permitting ontology-based *semantic* markup of named entities (http://ucsdbiolit.codeplex.com/). Routine use of these applications would enable authors to add both structural and semantic markup to articles with minimal effort.

Authors' raw numerical datasets are almost always held in spreadsheets. If requested to do so by journals, authors could easily submit these spreadsheets with manuscripts, for publication as downloadable Supporting Information files. Thus, given the correct tools and incentives, authors could contribute most of the data and create much of the metadata required for semantic publishing during the course of article writing, with marginal additional effort.

### Automated Named Entity Recognition: The Role of Text Mining

To enable textual semantic markup to be undertaken cost-effectively across the publishing world, an alternative to author markup at the time of writing is automated text mining applied to the submitted manuscript. In the biological domain, the BioCreAtIvE Challenge (Critical Assessment of Information Extraction systems in Biology) (http://biocreative.sourceforge.net/, http://www.biocreative.org/), now in its sixth year, has been very effective in catalysing a critical comparative evaluation of the capabilities of text mining and information extraction systems applied to the biological literature. A special issue of Genome Biology (http://genomebiology.com/supplements/9/S2), which contains 14 papers arising from BioCreAtIvE II, presents the state of the art.

There has already been considerable discussion in the literature concerning the potential role of text mining in extracting from full-text articles the information required to describe protein–protein interactions in Structured Digital Abstracts, and reciprocally for SDAs to assist full-text text mining [4], [5], [27]–[30]. Indeed, the current round of BioCreAtIvE, BioCreAtIvE II.5 (http://www.biocreative.org/news/chapter/biocreative-ii5/), is focusing on automated tools to replicate manual production of structured digital abstracts, using *FEBS Letters* as the dataset, blurring the distinction between authoring tools and post-submission analysis. It has been concluded that while current text-mining techniques are adequate for recognition of named entities (e.g., gene names), the extraction of relationships is a task for which information extraction tools still require additional development [30]. Our own experience in marking up the *PLoS NTDs* article shows that a measure of expert human supervision is likely to be required to avoid ambiguities.

In our testing of freely available semantic categorization services, we found the uBio Taxon Finder Web service for automatically finding taxonomic names within text (http://www.ubio.org/index.php?pagename=xml_services) to be highly accurate when applied to the text of our selected *PLoS NTDs* article, while the free Reuters OpenCalais automated text-mining service (http://www.opencalais.com/) was excellent at recognising persons, place names, and institutions, as might be expected, but performed very poorly for domain-specific biological terms.

Other more sophisticated text-mining and natural language processing tools are currently being developed to recognise textual instances and to link them automatically to domain-specific ontologies, and it is to be hoped that such developments at the interface between text mining and semantic technologies will in future facilitate automated markup of journal articles.

## Conclusions

The UK Government's 2006 Foresight Report (http://www.foresight.gov.uk/Infectious%20Diseases/E1_ID_Executive_Summary.pdf) concluded that the future threats from infectious diseases are at least as great as in the past century, with an undiminished rate of emergence of new diseases and increasing resistance being shown by many disease agents to antimicrobial drugs. Malaria and TB continue to be major global health problems; SARS, HIV-AIDS, West Nile fever, bluetongue, and avian influenza have recently presented new hazards; and bioterrorism presents potential threats.

Our motivation for undertaking this work was to demonstrate that it is possible to change the world in terms of semantic enhancements of research publications—both of papers and datasets—by simple application of existing Web technology. We have been particularly keen to demonstrate this for a paper relevant to infectious disease epidemiology, since it is clear that lives may depend upon the timely availability of reliable disease incidence data that permit predictions of the severity and spread of epidemics.

**Figure 10:**
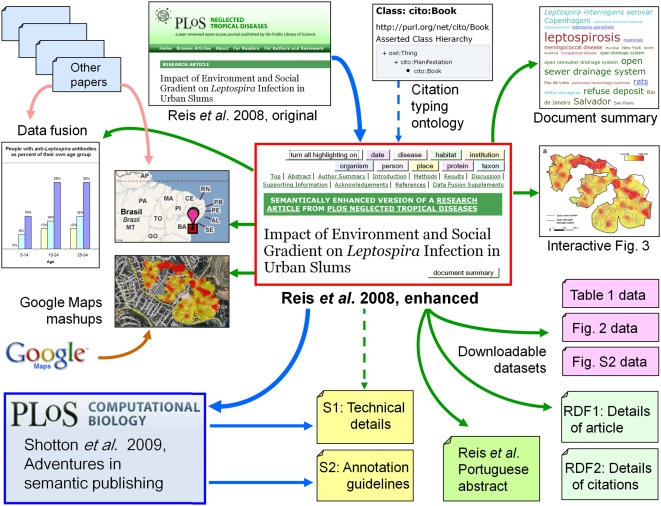
The ecosystem of published articles, documents, spreadsheets, data fusions, and machine-readable RDF data files resulting from our decision to apply semantic enhancements to the *PLoS NTDs* article by Reis et al. [12].

Our semantic enhancements of the single article by Reis et al. (2008) [12] have led to the creation of a whole “ecosystem” of articles, documents, spreadsheets, data fusions, and RDF files related to that original work, considerably more numerous than we anticipated when we started, in addition to the enhancement features contained within the *PLoS NTDs* article itself. Figure 10 presents a visual summary of these various outputs, including this *PLoS Computational Biology* paper, which we hope will be useful to readers in clarifying the different types of semantic enrichment created.

We solicit feedback from readers (by e-mail to david.shotton@zoo.ox.ac.uk) about the added value they perceive in these various enhancements and outputs, relative to the originally published version of the *PLoS NTDs* article; about ways in which our work could be improved, and additional enhancements readers would wish to see; and about how such semantic publishing can be moved from bespoke manual crafting to mainstream journal production. We hope this work will encourage authors, data producers, publishers, and information consumers to use the Web to its full potential for scientific publications.

## Supporting Information

Text S1 Shotton D, Portwin K (2009). Technical implementation of the semantic enhancements applied to Reis et al. (2008) Impact of environment and social gradient on Leptospira infection in urban slums. PLoS Neglected Tropical Diseases 2(4): e228.
(0.16 MB DOC)

Text S2 Portwin K, Shotton D (2009). Annotation guidelines: Heuristics applied while selecting terms for semantic markup from the text of Reis et al. (2008) Impact of Environment and Social Gradient on Leptospira Infection in Urban slums. PLoS Neglected Tropical Diseases 2(4): e228.
(0.05 MB DOC)
