# Supplementary material for: Adventures in Semantic Publishing: Exemplar Semantic Enhancements of a Research Article
Source: PLoS Comput Biol. 2009 Apr 17;5(4):e1000361. doi: 10.1371/journal.pcbi.1000361 (PMC2663789; doi:10.1371/journal.pcbi.1000361)
Supplement: Text S1 — Shotton D, Portwin K (2009). Technical implementation of the semantic enhancements applied to Reis et al. (2008) Impact of environment and social gradient on Leptospira infection in urban slums. PLoS Neglected Tropical Diseases 2(4): e228. (0.16 MB DOC) [file pcbi.1000361.s001.doc]

# Text S1

# Technical implementation of the semantic enhancements applied to Reis *et al*. (2008) Impact of environment and social gradient on *Leptospira* infection in urban slums. *PLoS Neglected Tropical Diseases* 2(4): e228.

# by David Shotton and Katie Portwin

# Image Bioinformatics Research Group, Department of Zoology, University of Oxford

# South Parks Road, Oxford OX1 3PS, UK

**Contents**

Introduction

Self-referencing information for this document

Copyright and license statement

Functional enhancements to the *PLoS NTD* article and their technical implementation

Within-document navigation

Provision of new hyperlinks

Highlighting of semantic terms

'Citations in context': the Supporting Claims Tooltip

Provenance information

Alternative language abstract

Provision of a document summary

Citation typing using CiTO, the Citation Type Ontology

Optional re-ordering of the reference list

Interactive figures

Actionable data from figures and table

Data fusion with information from other sources

Provision of machine-readable metadata

References

**Introduction**

Semantic enhancements were made by David Shotton, Katie Portwin, Graham Klyne and Alistair Miles, [Image Bioinformatics Research Group](http://ibrg.zoo.ox.ac.uk/), Department of Zoology, University of Oxford to the above-cited *PLoS Neglected Tropical Diseases* (*PLoS NTD*) article by Reis *et al*. (2008). The semantically enhanced version of that article was published on 3 September 2008 at [doi:10.1371/journal.pntd.0000228.x001](http://dx.doi.org/10.1371/journal.pntd.0000228.x001), and the paper by Shotton *et al*. (2009), for which this is Supporting Information S1, describes the full range of semantic enhancement applied to that Reis *et al*. (2008) article. This document provides a technical description of how those semantic enhancements were implemented. A separate document, Supporting Information S2 by Portwin and Shotton, describes the heuristics we applied when deciding which textual terms were to be assigned to the semantic classes highlighted in the text of the enhanced version of the Reis *et al*. (2008) article.

**Self-referencing information for this document**

*Citation*: Shotton D and Portwin K (2009) Technical implementation of the semantic enhancements applied to Reis *et al*. (2008) Impact of environment and social gradient on *Leptospira* infection in urban slums. *PLoS Neglected Tropical Diseases* **2**(4): e228.

This MS Word document forms Supporting Information S1 to Shotton, D., Portwin, K., Klyne, G. and Miles, A. (2009) Adventures in semantic publishing: exemplar semantic enhancement of a research article. *PLoS Computational Biology* (submitted for publication; DOI to be assigned).

It is also separately published as an HTML Web document associated with the enhanced paper itself at <http://dx.doi.org/10.1371/journal.pntd.0000228.x009>.

Corresponding author: David Shotton <[david.shotton@zoo.ox.ac.uk](mailto:david.shotton@zoo.ox.ac.uk)>.

**Copyright and license statement**

© 2009 David Shotton, University of Oxford. This document, the semantic enhancements we made to Reis *et al*., 2008, the enhanced version of that article, and the original article are all open-access publications distributed under the terms of the [Creative Commons Attribution License](http://creativecommons.org/licenses/by/2.5/), which permits unrestricted use, distribution, and reproduction in any medium, provided the work is attributed to the original authors and sources.

**Functional enhancements to the *PLoS NTD* article**

The purpose of the semantic enhancements applied to the *PLoS NTD* article by Reis *et al*. (2008) is described in the paper by Shotton *et al*. (2009), while a wider review of the current state and potential usefulness of semantic publication is given in a separate paper (Shotton, 2009).

The enhancements now visible at [doi:10.1371/journal.pntd.0000228.x001](http://dx.doi.org/10.1371/journal.pntd.0000228.x001) in the enhanced version of the *PLoS NTD* article by Reis *et al*. (2008) were developed incrementally over a period of about six weeks during the summer of 2008. A subversion (svn) repository was used to store versions of the enhanced article, and a wiki was employed to record our methods and experiences. The Cascading Style Sheet (CSS) and JavaScript files used in the final enhanced version of the selected PLoS NTD article are to be found at <http://purl.org/net/semanticpublication/pntd.0000228/enrichment.css> and <http://purl.org/net/semanticpublication/pntd.0000228/enrichment.js>. We used namespaces that are simple, relevant and widely used: DC, DC Terms, FOAF, PRISM (selected terms), FRBR, time and Geo. Where we could find no appropriate external ontology, as was the case for citation typing, we created one, the Citation Type Ontology (<http://purl.org/net/cito/>), described below. An explanation of the technical implementation of the various semantic enhancements now follows:

***Within-document navigation***

We moved the tabs required to activate the sectional navigation links between different section of the article into a non-scrolling link set at the top of the document, adding one additional link, ‘Data Fusion Supplements’, that takes the reader to an additional section at the end of the article where links to these data fusions are given. The non-scrolling nature of these internal link tabs is achieved via CSS:

<div class=\"highlighting-toolbar\">

.highlighting-toolbar {

position:fixed;

top:0pt;

(N.B. this works in Firefox only, falling back to being non-floating in Internet Explorer)

We retained all the other pre-existing in-text links in the published article: from authors' names to their institutional addresses; from in-text citations of the figures, table and references to the corresponding items; from the figure and table thumbnails to their original full-size versions in the original article's ‘slideshow’; and from the titles of Supplementary Figures S1 and S2 in the main text to their original downloadable versions.

***Provision of new hyperlinks***

We added Web hyperlinks:

1. to the home pages of the authors' academic institutions, and to their funding agencies,
2. to software suppliers, infectious disease research centres and government agencies cited in the article,
3. to Connotea and Delicious,
4. to the Creative Commons license for the enhanced work,
5. to the W3C XHTML/RDFa Web page validation service.

We added an enhancement citation text box which contains a link to our own Image Bioinformatics Research Group home page.

We added hyperlinked DOIs for 28 journal article references, and for the first few also provided exemplar links to PubMed and PubMed Central. For references lacking DOIs, we added direct hyperlinks where available.

All such links were implemented conventionally using anchor tags and href attributes, e.g.

<a href="http://creativecommons.org/licenses/by/2.5/">Creative Commons Attribution License</a>.

***Highlighting of semantic terms***

We provided semantic enhancements to the title, text and reference titles, in the form of optional coloured highlighting for textual instances of nine classes of textual entities: **date**, **disease**, **habitat**, **institution**, **organism** (English name), **person** (Proper name), **place**, **protein** and **taxon** (Linnaean genus or species Latin name), each class being associated with a particular colour. The default setting for viewing the enhanced article is to have no highlighting shown, but the reader can select to have all the highlighting turned on, or to have one or more selected classes of terms highlighted, these options being chosen using coloured selection buttons located in a non-scrolling button set at the top of the document.  Decisions as to which words to highlight were guided by a set of heuristics we developed, described in Portwin and Shotton (supporting information Text S2).

Words and phrases in the text were marked up inline in the HTML document with <span> tags and class attributes corresponding to their category, e.g.

“. . . urban health problem as <span class="habitat">slum settlements</span> have expanded worldwide . . .”

while the "highlighting on/off" feature was achieved via CSS, JavaScript and the Yahoo! User Interface (YUI) Library of utilities and controls for building richly interactive web applications, written in JavaScript, as shown:

1. The article HTML was wrapped in a containing DIV styled with _highlightoff classes:

“<div id="highlighting-container" class="disease_highlightoff habitat_highlightoff place_highlightoff...">"

2. Nested CSS styles were defined in the enrichment.css file as shown:

.habitat_highlighton .habitat {

background-color: #9BFC94

}

.habitat_highlightoff .habitat {

background-color: #DAFAD8

}

3. A button in the toolbar enabled the class highlighting to be switched on or off:

<button class=\"habitat\" onclick=\"highlight(\'habitat\')\">habitat</button>

4. A JavaScript function in the enrichment.js file was used to add and remove styles:

function highlight(terms){

// available styles

var styleOff = terms+'_highlightoff';

var styleOn = terms+'_highlighton';

// is it currently on or off?

var currentStyle=YAHOO.util.Dom.get('highlighting-container').className;

var on = (currentStyle.indexOf(styleOn)>-1 ? true : false);

// toggle

YAHOO.util.Dom.removeClass('highlighting-container', (on ? styleOn : styleOff));

YAHOO.util.Dom.addClass('highlighting-container', (on ? styleOff : styleOn));

}

5. The "turn all highlighting off" button and its corresponding function alloff work in a similar way.

6. The non-scrolling feature of the highlighting toolbar is achieved using the position:fixed feature in CSS, as described above for the navigation links described (N.B. this works in Firefox only, falling back to being non-floating in Internet Explorer).

Most of the highlighted semantic terms were given no external links. However, to illustrate the principal, power and usefulness of such links to external authorities and ontologies, each instance of an **organism** was given a live hyperlink to the hierarchical Linnaean classification of that species provided by uBio (<http://www.ubio.org/>), e.g.

<a href="http://www.ubio.org/browser/details.php?namebankID=1903523" class="organism">chickens</a>.

***The Supporting Claims Tooltip to permit 'Citations in Context'***

To illustrate the possibility of permitting key evidence from a cited article to be presented to the reader in the context of the initial in-text bibliographic citation, we implemented a **Supporting Claims Tooltip** for two citations of the same reference (a key paper by the same senior author) made in different contexts. This permits relevant statements from the cited reference to be displayed in a small 'hover box' when the reader hovers the mouse pointer over the relevant in-text reference citation. Tooltips showing short summaries of linked-to resources are not new, and are often used in contextual advertising. The novel feature in this work is that the linking occurs at the level of claims, the two Supporting Claims Tooltips we implemented for separate citations of the same referenced article returning distinct information relevant to the context of each citation. We call this service ‘Citations in Context’.

Thus, as can be seen in the enhanced article, for the first and the third citation of reference [6] in the Introduction, shown in the enhanced text thus: **[6]**, we provide different supporting claims in the two pop-up Supporting Claims Tooltips. These claims were selected manually after inspection of the context of the citation and the text of the cited article.

These Tooltips are initialised when the enhanced HTML document is loaded:

YAHOO.util.Event.onDOMReady(initTooltips());

enrichment.js:

function initTooltips(){

tt1 = new YAHOO.widget.Tooltip(

"tt1",

{

context:"tooltip_ref6_occ3",

text:document.getElementById("tooltip_ref6_occ3_body").innerHTML,

autodismissdelay:60000

}

);

. . . etc. for each tooltip, which is attached to an anchor (the red **[6]**):

<a id="tooltip_ref6_occ3" href="#pntd.0000228-Ko1">[6]</a> .

The tooltip content is given in a named element, e.g.:

<div id="tooltip_ref6_occ3_body" class="tooltip_body">

Albert I Ko et al. (1999) <b>"Urban epidemic of severe leptospirosis in Brazil"</b><br/><br/>

<b>Supporting claims:</b>

<ul>

<li><b>Results:</b><i>"..Severe flooding occurred during the heaviest period of rainfall between April 21 and April 27. The largest number of cases per week (39) was reported 2 weeks after this event...."</i></li>

<li><b>Results:</b><i>"Figure 2. Weekly cases of leptospirosis and rainfall in Salvador, Brazil, between March 10, and Nov 2, 1996"</i><br/>

<img width="100" height="100" src="http://www.sciencedirect.com/cache/MiamiImageURL/B6T1B-45X015F-D-8/0?wchp=dGLbVzb-zSkzV" alt="Reference [6] Occurrence (3) - Figure 2"/></li>

</ul>

</div> .

***Provenance information***

To each item relating to the original *PLoS NTD* article that we modified or published anew, we added statements detailing the provenance of the document and citing the original article to which it relates, as at the head of this document.

***Alternative language abstract***

We converted the Portuguese abstract from a downloadable Word file into a Web document, identified key semantic terms within it (e.g. galinhas (chickens)), and added buttons to permit the highlighting of these semantic terms, as in the main article. We assigned a DOI (<http://dx.doi.org/10.1371/journal.pntd.0000228.s003.x001>) to the Portuguese abstract, and moved the link to it to a position immediately following the English language abstract in the main article.

***Provision of a document summary***

We created a human-readable document summary (<http://dx.doi.org/10.1371/journal.pntd.0000228.x002>), accessed by clicking the Document Summary button immediately following the title of the enhanced *PLoS NTD* article. This contains six sections:

1. **Study summary**. A simple table, specifying the disease studied, its pathogenic causative agent, principal vector, and pathogen host; the number of subjects and controls involved in the study; the indicator of infection and the assay used to detect it; the name and location of the study site and the start and end dates of the study; and the purpose of the study and the study’s principal findings.
2. **Tag cloud**. A tag cloud, showing in alphabetical order the terms highlighted in the text of the article (with the exception of institutional and personal names), displayed in their appropriate highlighting colours and with sizes proportional to their frequency of occurance in the text.
3. **Tag trees**. Listings of these terms separated into their nine semantic classes, arranged, where appropriate, into informal hierarchies that we call **tag trees.**
4. **Infectious disease ontology terms**. Those terms relevant to the subject matter of the study by Reis et al. (2008) [12] that are present in the Infectious Disease Ontology (http://www.infectiousdiseaseontology.org) are presented as a simple list, in numerical order of their identifiers.
5. **Document statistics**. A simple set of document statistics, summarizing the number of authors, cited references, figures, supplementary figures and tables in the article.
6. **Citation analysis**. A simple numerical analysis of the frequency of reference citations in different parts of the document (Introduction, Methods and Discussion), both as numerical tables and as histograms.  The numerical data and histograms of this citation analysis were additionally made available as an Excel spreadsheet, downloadable from the Document Summary.

To implement the tag cloud, we first had to count the number of instances of each highlighted term, using /utils/Scrape.java. The following is the example output for the class **habitats**:

accumulated refuse *3

Atlantic rain forest *1

cities *3

hills *1

household *1

household environment *5

household property *2

households *14

open accumulated refuse *1

open drainage systems *1

open rainwater *1

Open rainwater drainage structures *1

open rainwater drainage system *1

open refuse deposit *2

open refuse deposits *2

open sewage and rainwater drainage systems *1

open sewer *9

open sewers *11

peri-domiciliary environment *1

refuse *2

refuse deposit *2

refuse deposits *6

The next task was to collapsed synonyms and plurals. For example, the terms 'refuse', 'accumulated refuse', 'open accumulated refuse', 'refuse deposit', 'refuse deposits', 'open refuse deposit' and 'open refuse deposits' were manually amalgamated into a single term, 'refuse deposit', with an appropriate weighting. This was undertaken manually.

Structuring the resulting terms into the hierarchical trees that we call **tag trees** was also undertaken manually. For example, ‘open rainwater drainage system’ was put as a child term of ‘open drainage system’. Implementation of these features was via the Cascading Style Sheet, thus:

***Colours***

.tagcloud .habitat{

color:#1A6F09;

}

<span class=\"tagcloud\">

<span class=\"habitat tagcloud2\">open drainage system</span><br/>

<span class=\"indent1 habitat tagcloud3\">open rainwater drainage system</span><br/>

***Indent***

.indent1{

position:relative;

left:50px;

}

<span class=\"tagcloud\">

<span class=\"habitat tagcloud2\">open drainage system</span><br/>

<span class=\"indent1 habitat tagcloud3\">open rainwater drainage system</span><br/>

***Size***

.tagcloud1 {

font-size:12pt;

}

<span class=\"tagcloud\">

<span class=\"habitat tagcloud2\">open drainage system</span><br/>

<span class=\"indent1 habitat tagcloud3\">open rainwater drainage system</span><br/>

Separate from this human-readable Document Summary, we provided a machine-readable RDF document information file in Notation3 format containing basic citation information about the article itself (see below).

***Citation typing using CiTO, the Citation Typing Ontology***

To provide a controlled vocabulary for describing and typing citations of other papers in the *PLoS NTD* article’s reference list, we developed **CiTO**, **the Citation Typing Ontology** (<http://purl.org/net/cito/>), and used this to type the references in the *PLoS NTD* article in three ways:

In using CiTO, each cited reference should be typed in three ways:

1. In terms of **the relationship between the citing work A** (i.e. Reis *et al*., 2008) **and the cited work B**, from the point of view of the citing work (i.e. Reis et al.’s *PLoS NTD* article), indicated in blue in the citation typed reference list (e.g. '*obtains background from*' indicates that A obtained background information from B, while '*uses method in*' indicates that A used a method described in B) (these are *Object Properties* in CiTO).
2. In terms of **the Nature or Type of the cited work B**, indicated in magenta (e.g. '*Research Paper*' indicates that B reports original research findings, while '*Review*' indicated that B is a scholarly review of others' research findings) (these are *Sub-classes of Work* in CiTO).
3. In terms of **the Manifestation of the cited work B**, indicated in red (e.g. '*Journal Article*' indicates that B is a journal article, while '*Print Document*' indicates that B is a print document that is not available online) (these are *Sub-classes of Manifestation* in CiTO).

In the enhanced *PLoS NTD* article, this citation typing is not displayed by default, but may be revealed, in the colours specified, by clicking the ‘Turn citation typing on’ button that immediately precedes the reference list. This feature is implemented as follows:

<button onclick="highlight('citationtype')">Turn citation typing

<span class="citationtypebuttonon">on</span>

<span class="citationtypebuttonoff">off</span>

</button>

<ol class="references" id="references">

For each reference the citation frequency and citation typing is encoded as follows:

<li id="ref1" class="ref citedfrequency4"><table><tr><td>1. </td><td><a id="pntd.0000228-United1"></a> <span class="authors">United Nations Human Settlements Programme</span> (2003) The challenge of <span class="habitat">slums</span>: Global report on human settlements <span class="date">2003</span>. London: Earthscan Publications Ltd.

<a href="http://www.unhabitat.org/downloads/docs/GRHS.2003.0.pdf" >Link</a>

<span class="citationtype">(CiTO: <span class="cito_relationship">obtains background from</span>, <span class="cito_type">Report</span>, <span class="cito_manifestation">Online Document</span>)</span>

</td></tr></table>

</li>

etc. for the subsequent references.

***Optional re-ordering of the reference list***

We added an array of buttons immediately after the References heading that gives the reader the ability to re-order the reference list in **alphabetical order**, by **publication year**, by **frequency of in-text citation**, or by **reference number** (i.e. the original published order).

The technical implementation of this re-ordering involves wrapping the existing ordered list of references in a container <ol id="references">, each reference being labelled with a numbered ID, e.g. <li id="ref1"> .

The re-ordering buttons call a JavaScript function giving an appropriately ordered list of reference ids, e.g. for sorting by year:

<button onclick="sort_references(new Array(20,40,2,19,21,22,23,24,39,50,52,10,30,37,51,3,5,7,14,27,32,46,17,44,1,8,15,33,34,36,16,13,31,47,9,28,43,45,48,4,11,18,29,38,6,26,35,25,42,49,41,12)) .

The JavaScript function removes the reference elements from the document object model (dom):

var container = document.getElementById('references')

while(container.hasChildNodes()){

container.removeChild(container.firstChild);

}

and then re-inserts them, in the new order:

for(n=0; n<order.length; n++){

container.appendChild(refElements[order[n]]);

} .

Note that the default styling for the re-ordered list – that the first item should be labelled 1 – is no longer appropriate. Rather, the references should keep their original numbers. Therefore it is necessary to remove the default styling:

.references LI {

list-style-type:none;

}

and add the numbers explicitly, e.g.:

<td>1. </td><td>United Nations Human Settlements Programme</span> (2003) The challenge of slums: Global report on human settlements...

For sorting by frequency of citation, new styling was required in order to present the references with font sizes proportional to their frequency of citation within the article. A reference LI class based on frequency was established:

<li id="ref2" class="ref citedfrequency3"$gt;

and a corresponding CSS entry was used, e.g.:

.references_citedfrequencyon .citedfrequency3{

font-size:16pt;

}

with a wrapper element:

<ol class="references" id="references">.

The ‘Sort by frequency of citation’ button calls JavaScript:

<button onclick="sort_references...;references_frequency_tagcloud_on()"

which adds a style class:

function references_frequency_tagcloud_on(){

YAHOO.util.Dom.addClass('references', 'references_citedfrequencyon');

} .

One additional aspect of Citations in Context , described above, also involves reference re-ordering: clicking on the selected reference citation in each instance in which the Supporting Claims Tooltip was implemented takes the reader, as expected, to that reference in the reference list at the end of the document.  However, in these particular cases the displayed references are slightly re-ordered so that the cited reference is immediately followed by the other references cited in the same context. That is implemented similarly. Clicking on the inline anchors results in re-ordering and grouping of the reference list, as follows:

The anchor tag calls sort_references with the appropriate two-dimensional array. Thus in the first instance, when reference **[6]** is cited with reference [7]:

<a

href="#pntd.0000228-Ko1"

onclick="sort_references(

new Array(

new Array(1,2,3,4,5),

new Array(6,7),

new Array(8,9,10,11,12,13,14,15,16,17,18,19,20,21,22,23,24,25,26,27,28,29,30,31,32,33,34,35,36,37,38,39,40,41,42,43,44,45,46,47,48,49,50,51,52)

)

);">

[6]</a> .

Sort-references builds the nested OLs:

var ol = document.createElement("ol"); etc.

In the second selected instance, when reference **[6]** is cited with references [11]-[18], the arrays are altered to become:

new Array(

new Array(1,2,3,4,5),

new Array(6,11,12,13,14,15,16,17,18),

new Array(7,8,9,10, 19,20,21,22,23,24,25,26,27,28,29,30,31,32,33,34,35,36,37,38,39,40,41,42,43,44,45,46,47,48,49,50,51,52)

)

***Interactive figures***

As an example of what can be done to assist the reader in quickly obtaining a fuller understanding of the results obtained, we created an enhanced interactive version of Figure 3 from the *PLoS NTD* article, that permits the user to drag and drop the individual panels of the figure, superimposing them on one another at will. To enable such overlay to be useful, Panels A, B and C have been made semi-transparent, and Panel D has been constrained to lie behind the other panels when they are dragged over it. This was made possible using CSS, JavaScript and the Yahoo! User Interface (YUI) Library of utilities and controls; specifically:

(a) the dragdrop function:

<link rel="stylesheet" type="text/css" href="http://yui.yahooapis.com/2.5.2/build/fonts/fonts-min.css" />

<script type="text/javascript" src="http://yui.yahooapis.com/2.5.2/build/yahoo-dom-event/yahoo-dom-event.js"></script>

<script type="text/javascript" src="http://yui.yahooapis.com/2.5.2/build/dragdrop/dragdrop-min.js"></script>

<link rel="stylesheet" type="text/css" href="overlaystyle.css" />

<link rel="stylesheet" type="text/css" href="plos.css" />

(b) setting up the initial array of Figure panels:

<div class="tilegroup">

<span class="tilegrouprow">

<img src="a.png" alt="A" class="op tile" style="z-index:30" id="a"/>

<img src="b.png" alt="B" class="op tile" style="z-index:20" id="b"/>

</span>

<span class="tilegrouprow">

<img src="c.png" alt="C" class="op tile" style="z-index:10" id="c"/>

<img src="d.png" alt="D" class="tile" style="z-index:0" id="d"/>

</span>

<span class="tilegrouprow">

<img src="e.png" alt="E" class="tile" style="z-index:40" id="e"/>

<img src="f.png" alt="F" class="tile" style="z-index:50" id="f"/>

</span>

</div>

and (c) activating the drag and drop:

<script type="text/javascript">

(function() {

var dda, ddb, ddc, ddd, dde, ddf;

YAHOO.util.Event.onDOMReady(function() {

dda = new YAHOO.util.DD("a");

ddb = new YAHOO.util.DD("b");

ddc = new YAHOO.util.DD("c");

ddd = new YAHOO.util.DD("d");

dde = new YAHOO.util.DD("e");

ddf = new YAHOO.util.DD("f");

});

})();

</script>

***Actionable data from figures and table***

To permit readers to obtain numerical data from the *PLoS NTD* article in actionable form, as opposed to the image formats provided originally by PLoS, we published downloadable Excel spreadsheets containing the raw data from three of the article’s figures kindly provided to us by the authors of Reis *et al*., 2008, supplemented by identification and provenance information, and provided hyperlinks to these from the figure legends within the article, using the following DOIs:

Data from Table 1: <http://dx.doi.org/10.1371/journal.pntd.0000228.t001.x001>;

Data from Fig. 2: <http://dx.doi.org/10.1371/journal.pntd.0000228.g002.x001>;

Data from Supplementary Figure S2: <http://dx.doi.org/10.1371/journal.pntd.0000228.s002.x001>.

***Data fusion with information from other sources***

We created five data fusions (‘mashups’) with relevant information from elsewhere:

***A simple geospatial data fusion with Google Maps***

One figure from the article, Fig. 3c. showing the distribution of risk of contracting Leptospirosis within the study site, was integrated with Google Maps so that the study site described in the article could be seen in the context of the street plan or satellite photograph of the city of Salvador, Brazil. The DOI URL for this data fusion is <http://dx.doi.org/10.1371/journal.pntd.0000228.g003.x001>.

KML is a file format used to display geographic data in an Earth browser such as Google Maps or Google Earth. It uses a tag-based structure with nested elements and attributes based on the XML standard. For our data fusion, a machine-readable KLM metadata file containing the geospatial coordinates for the selected figure was created and stored at <http://purl.org/net/semanticpublication/pntd.0000228/enhanced/Fig3-map-overlay-kml>. Its file name is passed as a parameter to Google Maps, which handles all the interactive features in this data fusion, in the following URL: <http://maps.google.com/maps?q=http://purl.org/net/semanticpublication/pntd.0000228/enhanced/Fig3-map-overlay-kml>. The contents of the KLM file itself are as follows:

<kml>

<GroundOverlay>

<name>

Spatial distribution of subjects with Leptospira antibodies.

Figure 3(C) from:

Impact of Environment and Social Gradient on Leptospira Infection in Urban Slums (2008) Reis et al., PLoS Negl Trop Dis 2 (4): e228.

</name>

<description>

<a href="http://dx.doi.org/10.1371/journal.pntd.0000228.g003">

http://dx.doi.org/10.1371/journal.pntd.0000228.g003

</a>

</description>

<Icon>

<href>

http://imageweb.zoo.ox.ac.uk/pub/2008/geomashupdev/overlay/heatmap_leptosipra_paudalima_salvador_brazil.png

</href>

</Icon>

<LatLonBox id="khLatLonBox751">

<north>-12.92078887426644</north>

<south>-12.928554379691054</south>

<east>-38.430634088134766</east>

<west>-38.441233363342285</west>

<rotation>0</rotation>

</LatLonBox>

</GroundOverlay>

</kml> .

***Geospatial data fusion together with disease incidence data for the city of Salvador***

The same Fig. 3c. from Reis *et al*. (2008) was integrated with Google Maps together with another map showing Leptospirosis incidence data for the city of Salvador by census district, published in a previous article by the same authors (Ko et al., 1999. The Lancet 354 (9181) (<http://dx.doi.org/10.1016/S0140-6736(99)80012-9>)). For this purpose, a second similar KLM file containing the geospatial coordinates for the two selected figures was created, stored at <http://purl.org/net/semanticpublication/pntd.0000228/enhanced/Fig3-incidence-kml>, and used in the same way. The DOI URL for this second data fusion is <http://dx.doi.org/10.1371/journal.pntd.0000228.g003.x002>. Note the slight misalignment of the 1999 map with Google Maps, which we have not attempted to correct.

***Geospatial localization of the study sites of several independent investigations of leptospirosis***

In the third data fusion, the geospatial locations of the study sites of several independent investigations of leptospirosis, including the one reported in the selected *PLoS NTD* article, were identified, and their longitudes and latitudes determined, permitting these locations to be identified on a world map using Google Maps. The KLM file for these study site locations and the citations of the articles describing them is recorded at <http://purl.org/net/semanticpublication/pntd.0000228/enhanced/leptospira-field-studies-kml>, and the DOI URL for the data fusion is <http://dx.doi.org/10.1371/journal.pntd.0000228.x006>. The Salavdor study site described by Reis *et al*., 2008 is indicated by the red square, while the additional study sites are indicated by the inverted-droplet shaped ‘location pins’. Clicking on any one of the symbols brings up a popup display showing the citation data for the relevant article.

In all three of the above cases, the overlaid data can be interactively removed or added back to the Google Maps display simply by unchecking or rechecking the check boxes in the panel to the left of the map itself. The zoom and scroll features of the data fusion are additional functionalities intrinsic to Google Maps.

***Geo*temporal *localization of these same study sites on the world map equipped with a time slider***

The fourth data fusion resembles the third, except that we equipped the map display with a time slider along the right margin, such that the locations of study sites are only displayed on the map if the publication dates of the articles describing them fall within the set temporal range. Our prototype has several limitations: (i) it uses article publication dates rather than the dates when the reported field studies were actually undertaken; (ii) the temporal resolution is at present only to the nearest year; and (iii) there is a subtle JavaScript bug that we have not yet isolated that causes the location pins and the pop-up infoboxes to be somewhat elongated in shape. Nevertheless, this data fusion serves to show the potential power of spatio-***temporal*** mapping.

***Serological data fusion across publications***

To illustrate the usefulness of data fusions that do *not* involve Google Maps, data showing the age distribution of disease incidence from Reis *et al*. (2008) were combined with similar data from a previous study by the same research group, Maciel *et al*. (2008), and re-plotted side by side in histograms to permit direct comparison.

Reis *et al*. (2008) looked at the occurrence of anti-*Leptospira* antibodies in a large representative population from a single slum community, Pau da Lima, in Salvador, Brazil. Maciel *et al*. (2008) looked at the occurrence of anti-*Leptospira* antibodies among healthy members of a few ‘index’ households of hospitalized patients with acute leptospirosis scattered across nineteen Salavador slums, and in neighbouring control households.

Both studies use a microagglutination test to detect anti-*Leptospira sp.* antibodies in the blood of subjects, and took the presence of such antibodies as evidence of prior exposure to these spirochete bacteria. This data fusion concerns the **age distribution of subjects** showing such immune responses to *Leptospira sp.* of any serotype.

Further details are given in the data fusion document itself, a downloadable Excel spreadsheet that is to be found at <http://dx.doi.org/10.1371/journal.pntd.0000228.x008>.

***Provision of machine-readable metadata***

We provided three types of machine-readable metadata concerning the Reis *et al*. (2008) article:

***RDFa***

Within the enhanced Reis *et al*. article (<http://dx.doi.org/10.1371/journal.pntd.0000228.x001>), we used RDFa to embed the article's self-referencing and provenance metadata.

***Notation3***

In addition, we published an expanded version of the article's self-referencing and provenance metadata, including the article's English language abstract, as a separate RDF document in Notation3 format (<http://dx.doi.org/10.1371/journal.pntd.0000228.x003>).

Finally, we published the full typed citation list from the *PLoS NTD* article in a second RDF Notation3 document (<http://dx.doi.org/10.1371/journal.pntd.0000228.x004>).

***References***

Ko AI, Reis MG, Ribeiro Dourado CM, Johnson WD Jr and Riley LW (1999). Urban epidemic of severe leptospirosis in Brazil. Salvador Leptospirosis Study Group. *Lancet* 354: 820–825. <http://dx.doi.org/10.1016/S0140-6736(99)80012-9>.

Maciel EAP, de Carvalho ALF, Nascimento SF, de Matos RB, Gouveia EL, Reis, MG and Ko, AI (2008). Household transmission of *Leptospira* infection in urban slum communities. *PLoS Neglected Tropical Disease* 2(1): e154. <http://dx.doi.org/10.1371/journal.pntd.0000154>.

Reis RB, Ribeiro GS, Felzemburgh RDM, Santana FS, Mohr S, Melendez AXTO, Queiroz A, Santos AC, Ravines RR, Tassinari WS, Carvalho MS, Reis MG and Ko AI (2008). Impact of environment and social gradient on *Leptospira* infection in urban slums. *PLoS Neglected Tropical Disease* 2(4): e228. <http://dx.doi.org/10.1371/journal.pntd.0000228>.

Shotton D (2009) Semantic Publishing: the coming revolution in scientific journal publishing. Learned Publishing 22: 85-94. <http://dx.doi.org/10.1087/2009202> (available online from mid-March 2009). Preprint available at

<http://purl.org/net/semanticpublication/Shotton_Semantic_publishing_evaluation.pdf>.

Shotton, D., Portwin, K., Klyne, G. and Miles, A. (2009) Adventures in semantic publishing: exemplar semantic enhancement of a research article. *PLoS Computational Biology* (submitted for publication; DOI to be assigned). Preprint available at <http://purl.org/net/semanticpublication/Shotton_et_al_PLoS_enhancement_report.pdf>.

/end
