# Supplementary material for: Adventures in Semantic Publishing: Exemplar Semantic Enhancements of a Research Article
Source: PLoS Comput Biol. 2009 Apr 17;5(4):e1000361. doi: 10.1371/journal.pcbi.1000361 (PMC2663789; doi:10.1371/journal.pcbi.1000361)
Supplement: Text S2 — Portwin K, Shotton D (2009). Annotation guidelines: Heuristics applied while selecting terms for semantic markup from the text of Reis et al. (2008) Impact of Environment and Social Gradient on Leptospira Infection in Urban slums. PLoS Neglected Tropical Diseases 2(4): e228. (0.05 MB DOC) [file pcbi.1000361.s002.doc]

# Text S2

# Annotation Guidelines

# Heuristics applied while selecting terms for semantic mark-up from the text of Reis *et al*. (2008) Impact of Environment and Social Gradient on *Leptospira* Infection in Urban slums, *PLoS Neglected Tropical Diseases* 2(4): e228

# by Katie Portwin and David Shotton

#

# Image Bioinformatics Research Group, Department of Zoology, University of Oxford

# South Parks Road, Oxford OX1 3PS, UK

**Contents**

Introduction

Self citation information for this document

Copyright and license statement

Background

Heuristics used for semantic mark-up

**Introduction**

Semantic mark-up of the text of the cited *PLoS Neglected Tropical Diseases* (*PLoS NTD*) article by Reis *et al*. (2008) was implemented manually by Katie Portwin and David Shotton, [Image Bioinformatics Research Group](http://ibrg.zoo.ox.ac.uk/), Department of Zoology, University of Oxford. The semantically enhanced version of that article was published on 3 September 2008 at [doi:10.1371/journal.pntd.0000228.x001](http://dx.doi.org/10.1371/journal.pntd.0000228.x001), and the paper by Shotton *et al*. (2009), for which this is Supporting Information S2, describes the full range of semantic enhancement applied to that Reis *et al*. (2008) article. A separate document, Supporting Information S1 by Shotton and Portwin, describes the technical implementation of those semantic enhancements, while this document describes the heuristics we applied when deciding which textual terms were to be assigned to the semantic classes highlighted in the text of the enhanced version of the article.

**Self-citation information for this document**

Portwin K and Shotton D (2009). Annotation Guidelines: Heuristics applied while selecting terms for semantic mark-up from the text of Reis *et al*. (2008) Impact of Environment and Social Gradient on *Leptospira* Infection in Urban slums, *PLoS Neglected Tropical Diseases* **2**(4): e228.

This MS Word document forms Supporting Information S2 to Shotton, D., Portwin, K., Klyne, G. and Miles, A. (2009) Adventures in semantic publishing: exemplar semantic enhancement of a research article. *PLoS Computational Biology* (submitted for publication; DOI to be assigned).

It is also separately published as an HTML Web document associated with the enhanced paper itself at <http://dx.doi.org/10.1371/journal.pntd.0000228.x010>.

Corresponding author: David Shotton <[david.shotton@zoo.ox.ac.uk](mailto:david.shotton@zoo.ox.ac.uk)>.

**Copyright and license statement**

© 2009 David Shotton, University of Oxford. This document, the semantic enhancements we made, the enhanced version of the article and the original article are all open-access publications distributed under the terms of the [Creative Commons Attribution License](http://creativecommons.org/licenses/by/2.5/), which permits unrestricted use, distribution, and reproduction in any medium, , provided the work is attributed to the original authors and sources.

**Background**

To enable semantic mark-up of text to be applied cost-effectively in a journal publishing environment, it will be necessary to automate it. Sophisticated text mining and natural language processing tools are currently being developed to recognise textual instances and link them automatically to domain-specific ontologies. However, our own experience in marking up the text of the chosen *PLoS NTD* article by Reis *et al*. (2008) clearly showed the requirement for human intervention. For example, we wished to record 'slums' and 'slum environments' as types of habitat in which the disease leptospirosis was likely to occur. However, blindly marking up every occurance of phrases in which the word 'slum' appeared was not appropriate, since a 'slum dweller' is clearly a person, not a habitat. To guide our mark-up, we developed the following set of simple heuristics that may be of assistance to others undertaking similar work.

**Heuristics for semantic mark-up**

We provided semantic enhancements to the title, abstracts and text of the *PLoS NTD* article and to the titles of the cited references in its reference list, in the form of optional coloured background highlighting, by marking up textual instances of nine classes of entities: **date**, **disease**, **habitat**, **institution**, **organism** (English name), **person (**a person's proper name**)**, **place**, **protein** and **taxon** (i.e. Linnaean genus or species Latin name), each class being associated with a particular colour.  In the following explanations, members of these classes are called ‘controlled terms’, and commonly occurring phrases that it would not be sensible to highlight are called ‘stop words’.

The heuristics we developed when deciding whether or not to apply highlighting to an occurrence of a particular term are as follows:

1. **Adjectival use of controlled terms**, e.g. 'slum dweller', '*Leptospira* antibodies', '*Leptospira* transmission', 'Mumbai slums', where the nouns 'slum', '*Leptospira*' and 'Mumbai' are themselves controlled terms:

- If the noun is a controlled term, treat as a single phrase and mark up according to the classification of the noun, e.g. '*Leptospira* antibodies' is a type of antibody, and hence a protein.
- If the noun is not a controlled term, do not mark up the phrase, e.g. 'slum dweller' and '*Leptospira* transmission' are not marked, since 'dweller' and 'transmission' are not controlled terms.
- However, where the noun is a controlled term and its qualifying adjective is a proper name that is also a controlled term, mark up each separately, e.g. for 'Mumbai slums', mark up 'Mumbai' as a place and 'slums' as a habitat.

     2.    **Stop words**, e.g. the occurrence of the noun 'disease', or of any other class name:

- Do not mark up, unless their qualification by a preceding adjective makes them more meaningful.  Thus 'disease' is not marked up, but 'childhood disease' is marked up as a type of disease.

    3.    **Ambiguous terms**, e.g. 'household' (which is used in the *PLoS NTD* article to mean either a physical house or a social group of persons):

- Infer the meaning from the context, e.g. for 'chickens in households', 'households' means physical buildings providing habitats, and is marked up; but in the phrase 'households raise chickens', 'households' mean social groups, and is not.

    4.    **Long phrases**, e.g. 'the sanitation infrastructure where slum inhabitants reside':

- Do *not* mark up the whole phrase if it is longer than three words.  While it is tempting to do so, one must draw the line!

Variations in sentence structure between language lead to interesting differences.  In the Conclusion of the [English Language Abstract](http://dx.doi.org/10.1371/journal.pntd.0000228.x001#abstract0) of our selected *PLoS NTD* article ([http://dx.doi.org/10.1371/journal.pntd.0000228.x001#abstract0](http://dx.doi.org/10.1371/journal.pntd.0000228.x001#abstract0)), the phrase 'slum residents' is not marked up, for the reason given above, since the word 'slum' is used adjectivally.  However, in the [Portuguese language abstract](http://dx.doi.org/10.1371/journal.pntd.0000228.s003.x001) (<http://dx.doi.org/10.1371/journal.pntd.0000228.s003.x001>), this phrase is translated 'residentes de favelas', since Romance languages have no compound noun formations, and so in this case the noun 'favela' (meaning slum or shanty town) *is* marked up as a habitat.

***References***

Reis RB, Ribeiro GS, Felzemburgh RDM, Santana FS, Mohr S, Melendez AXTO, Queiroz A, Santos AC, Ravines RR, Tassinari WS, Carvalho MS, Reis MG and Ko AI (2008). Impact of environment and social gradient on *Leptospira* infection in urban slums. *PLoS Neglected Tropical Disease* 2(4): e228 ([doi:10.1371/journal.pntd.0000228](http://dx.doi.org/10.1371/journal.pntd.0000228)).

Shotton, D., Portwin, K., Klyne, G. and Miles, A. (2009) Adventures in semantic publishing: exemplar semantic enhancement of a research article. *PLoS Computational Biology* (submitted for publication; DOI to be assigned). Preprint available at <http://purl.org/net/semanticpublication/Shotton_et_al_PLoS_enhancement_report.pdf>.

/end
